# Structural and epistatic regulatory variants cause hallmark white spotting in cattle

**DOI:** 10.1126/sciadv.adt5913

**Published:** 2025-11-14

**Authors:** Swati Jivanji, Emma Wilkinson, Lijing Tang, Kathryn M. Tiplady, Anna Yeates, Chad Harland, Charlotte Gray, Christine Couldrey, Gemma Worth, Isabelle Gamache, Jade Desjardins, John A. A. Tabares, Nobuko Yamanaka, Lorna McNaughton, Louise Brennan, Marie-Pier Cloutier, Mitra Cowan, Renae Ellison, Tony Fransen, Tracey Monehan, Richard J. Spelman, Russell G. Snell, Carole Charlier, Yojiro Yamanaka, Dorian Garrick, Richard Mort, Mathew D. Littlejohn

**Affiliations:** ^1^ Livestock Improvement Corporation, Newstead, Hamilton, New Zealand.; ^2^ AL Rae Centre for Genetics and Breeding, School of Agriculture and Environment, Massey University, Hamilton, New Zealand.; ^3^ School of Biomedical and Life Sciences, Lancaster University, Lancaster, UK.; ^4^ Unit of Animal Genomics, GIGA and Faculty of Veterinary Medicine, University of Liège, Liège, Belgium.; ^5^ McGill Integrated Core of Animal Modeling, McGill University, Montreal, Canada.; ^6^ Institute of Systems, Molecular and Integrative Biology, University of Liverpool, Liverpool, UK.; ^7^ School of Biological Sciences, University of Auckland, Auckland, New Zealand.; ^8^ Goodman Cancer Institute, Department of Human Genetics, McGill University, Montreal, Canada.

## Abstract

Despite being one of the most iconic and immediately recognizable traits in domestic cattle, the variants underpinning the white-spotted coat pattern of Holstein-Friesian and related breeds remain uncharacterized. Here, we report two variants modulating these effects, comprising intronic and long-distance–acting regulatory variants of the *MITF* and *KIT* genes. We confirm causality through “Holsteinized” mouse models edited for these alleles and show that these variants are likely responsible for spotting traits in other bovine breeds. These effects include epistatic impacts on other bovine coat patterns, such as fine-scale speckling, “black socks,” and reversal of the otherwise dominant, “white-face” trait characteristic of Hereford cattle.

## Teaser

A genetic exploration of how the Holstein got its spots.

## INTRODUCTION

Notable and unique coat patterns are a defining feature of domestic animal breeds. These traits are some of the earliest selected by humans, with our long-standing fascination with unusual coat patterns recorded in Neolithic and Paleolithic rock paintings of livestock (*1*, *2*). A variety of such patterns distinguishes modern breeds, with examples in cattle including the belted coats of Galloways, the “white-face” of Herefords and Simmentals, color sidedness in White Parks, and the white-spotted coat of Holstein-Friesians (HFs). With the exception of the latter, the variants underpinning all of these examples are now known (*3*–*6*), making white spotting one of the last breed-defining coat patterns yet characterized at the molecular level (*7*). This may be partially due to the trait showing an oligogenic architecture. Rather than being determined by a single Mendelian-effect variant, the degree of coat spotting appears to be determined through the aggregate effects of several major-effect quantitative alleles—and many more small ones (*8*, *9*). We previously reported mapping of the two largest-effect loci underlying coat spotting in a population of ~3000 HF, Jersey, and crossbred dairy cattle (*9*). Using imputed sequence data, one of these signals highlighted a candidate causative variant in a conserved intronic region of the *MITF* gene (*9*). However, no such candidate could be identified for the other, largest-effect locus, instead presenting a dispersed signal that broadly located to the *KIT* locus on chromosome 6.

Here, we aimed to definitively characterize these two loci as the major effects underpinning hallmark white spotting in HF dairy cattle. Using a combination of short- and long-read sequencing, we identify a noncoding structural variant on chromosome 6 that accounts for this effect. We investigate variant causality through cell models and experimental knockout of the homologous sequence in mice and show that the variant is carried by other typically white-patterned cattle breeds. Genome-edited mouse models also confirm the causality of the previously highlighted *MITF* intronic single-nucleotide polymorphism (SNP) as underlying the other major white spotting quantitative trait locus (QTL), where we further detail peculiar epistatic effects between this variant and other major coat-patterning loci.

## RESULTS

### Identification and characterization of a candidate structural variant at the *KIT* locus

Our previous genome-wide association study (GWAS) of white spotting highlighted two major-effect QTL each contributing >10% per-allele increases in spotting (*9*). For the chromosome 6 effect, this locus presented an unusually broad interval, potentially implicating a structural variant not well represented by the imputed short-read sequence data used for association mapping. To this end, we manually evaluated sequence alignments comprising a 20-Mbp interval encompassing the previous chromosome 6 GWAS signal. This analysis compared animals with contrasting genotypes for the rs463810013 SNP, a variant immediately adjacent the presumed causative gene *KIT* and the second most highly associated variant from GWAS (*9*). Discordant read pairs and possible spurious mapping were observed in cattle carrying the “solid colored” rs463810013 allele, highlighting split reads that mapped either side of the protein-coding sequence of *KIT* (fig. S1). Soft-clipped reads were observed in both upstream and downstream map locations (fig. S1), although mismatched sequences did not appear to be present elsewhere in the ARS-UCD1.2 reference genome (Materials and Methods). These observations suggested the presence of a structural variant of unknown composition overlapping the *KIT* locus. Although the structure of this variant was unclear, classification of 152 genome-sequenced individuals based on mismatched read status suggested that the variant was strongly linked to the QTL signal [coefficient of determination (*R*^2^) > 0.75 with rs463810013; table S1].

**Fig. 1.**
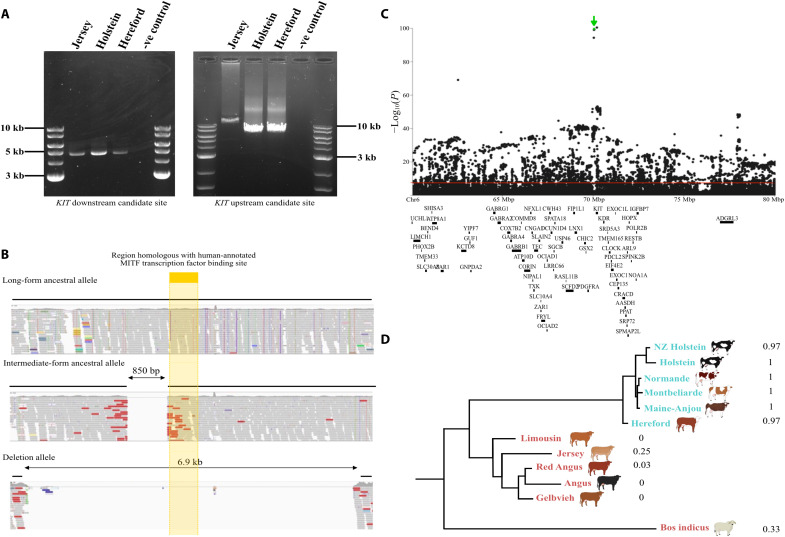
Molecular and bioinformatic characterization of candidate structural variants at the *KIT* locus. (**A**) Amplicons from PCR targeting the downstream candidate site (Chr6:70,394,382-70,399,130 bp; left) and upstream candidate (Chr6:70,048,910-70,055,246 bp; right) sites in a Jersey, Holstein, and Hereford sample. (**B**) Short-read sequence data mapped to the modified reference genome, showing the long-form ancestral allele (top), intermediate-form ancestral allele (middle), and deletion allele (bottom). Sequence homologous with the human-annotated MITF transcription factor binding site is shown in yellow. (**C**) Association results for proportion of white spotting on the coat with a 20-Mbp interval of sequence-resolution genotypes. The *KIT* structural variant (highlighted in green) had *P* value of 2.06 × 10^−100^, and the red line indicates the significance threshold *P* = 5 × 10^−8^. (**D**) Phylogenetic tree for typically spotted (blue) and nonspotted (red) cattle based on a 10-kbp region centered on the *KIT* structural variant region (Chr6:70,051,190-70,061,190 bp on the bespoke reference genome), with *B. indicus* included as an outgroup. The mash-based tree was constructed using sketch sizes of *s* = 1000 and *k*-mer sizes of *k* = 21.

To characterize this variant, we used polymerase chain reaction (PCR) and long-read sequencing in three Jersey bulls, three HF bulls, and two Hereford bulls selected to represent alternate genotypes of rs463810013 (table S2). Long-range PCR and Nanopore sequencing targeting a 4.7-kbp region encompassing the downstream candidate site (Chr6:70,394,382-70,399,130 bp) did not reveal any structural differences between bulls. However, amplification targeting a 6.3-kbp region encompassing the upstream candidate site (Chr6:70,048,910-70,055,246 bp) yielded a ~13-kbp amplicon in two Jersey bulls and an amplicon of expected size in all other bulls (Fig. 1A). These expanded sequences contained a 6948-bp insertion that was absent from the reference genome and flanked by two near-identical 42-bp bovine retrotransposable element repeat fragments (RTE-BovB). Notably, this repeat sequence was also present at the downstream candidate site, suggesting that all discordant short-read data may have represented mapping artifacts caused by the absence of the variant in the reference assembly. To test this hypothesis, we generated a modified reference genome containing the 6.9-kbp insertion and remapped 1126 cattle with short-read genome sequence data (Materials and Methods). Critically, these genomes lacked the discordant mapping shown in genomes mapped to the original ARS-UCD1.2 assembly, highlighting the candidate 6.9-kbp structural variant as likely responsible for all mapping anomalies at the *KIT* locus. These realignments also revealed two structural haplotypes of the insertion variant, comprising a long (6.9 kbp) and intermediate (~6 kbp) form of the same sequence (Fig. 1B). These alleles were largely absent from purebred HF and Hereford animals and presented frequencies of 0.62 and 0.13, respectively, in Jersey animals (table S3).

### The candidate structural variant is an evolutionarily conserved ancestral allele

Given that the ARS-UCD1.2 genome assembly represents a Hereford animal, and both HF and Hereford cattle present white on the coat and lack the structural alleles, we considered the “insertion” variants as the likely ancestral, solid-colored forms. In this context, the HF and Hereford allele represents the deleted state, so we hereafter refer to this allele as a deletion. This deleted noncoding sequence locates a considerable distance from the *KIT* gene, ~114-kbp upstream of exon 1 (NM_001166484.1). However, segments of this sequence appear highly conserved across various mammals, including human, mouse, and dog (fig. S2). Micro-C data from human (*10*) and mouse (*11*) embryonic stem cell lines also show that the variant sits within a ~700-kbp topologically associated domain that appears structurally conserved between species (fig. S2). Some sequences within the bovine ancestral allele are profoundly conserved, with one ~200-bp nested sequence near contiguous to human—presenting higher average nucleotide conservation than bovine and human *KIT* exonic sequences (94.2% versus 88.9% identity, respectively; fig. S2). Visualization of the human region via the UCSC Genome Browser (*12*) shows that this same conserved segment bears a distal enhancer-like signature (*13*) and contains hundreds of chromatin immunoprecipitation sequencing (ChIP-seq)–derived regulatory annotations (*14*). These include MITF transcription factor binding sites encompassing Chr4:54,571,230-54,571,627 bp on the GRCh38/hg38 reference assembly. Given the critical role of *MITF* in melanocyte biology (*15*) and its status as the gene underlying the other major-effect white spotting locus identified from GWAS (*9*), we considered knockout of this sequence as the likely mechanism of the *KIT* QTL. Notably, this sequence was present in both long- and intermediate-form ancestral alleles (Fig. 1B).

### Association analysis between *KIT* noncoding deletion haplotypes and white spotting

To test whether the *KIT* upstream deletion was associated with white spotting in New Zealand (NZ) dairy cattle, we imputed the long, intermediate, and deletion alleles into the population of 2967 mixed breed cattle with preexisting coat phenotypes (*9*). These alleles were represented as a single triallelic variant, with the imputation reference dataset consisting of genotypes manually scored from the 1126 sequence realignments based on our modified genome assembly (Materials and Methods).

We performed several association analyses to contrast the effects of the long, intermediate, and deletion alleles. Since the highly conserved, MITF binding site is present on both the long- and intermediate-form ancestral alleles, our first analysis merged these haplotypes for comparison with the deletion allele. Association analysis was conducted in conjunction with 152,071 other sequence variants imputed in the 20-Mbp interval previously shown to capture the white spotting QTL (*9*). The deletion ranked among the most highly associated variants for white spotting (*P =* 2.06 × 10^−100^ versus *P* = 3.21 × 10^−101^ for the lead variant Chr6 g.70210094A>C rs285773341; Fig. 1C) and was estimated to increase white spotting on the coat by 11.9 ± 0.2% per allele. When the deletion was fitted as a fixed effect in the association model, the significance of most variants in the 20-Mbp interval was lost (smallest *P* = 8.48 × 10^−11^ for Chr6 g.70343862A>T rs109258078). While some residual signal remained, we observed a similar result when fitting the top-associated variant Chr6 g.70210094A>C, highlighting the same Chr6 g.70343862A>T SNP as the new top variant (*P =* 7.64 × 10^−11^). While this might suggest the existence of an alternative biallelic variant not represented in our dataset, we considered this more likely to represent an additional *KIT* QTL, given that the variant appeared to be genetically independent of both Chr6 g.70210094A>C and the *KIT* structural variant (*R*^2^ ~ 0.02; table S4). Further association analysis comparing the deletion allele to either long or intermediate forms of the ancestral alleles similarly showed near-top ranked association for the deletion variant (fig. S3). Comparison of effects between long- and intermediate-form ancestral haplotypes did not reveal significant differences between these alleles (fig. S3). These findings support the hypothesis that the long and intermediate haplotypes are functionally equivalent, although the number of animals contrasting these alleles was limited (*N* = 85).

### The *KIT* noncoding deletion segregates in other breeds

Although HF cattle are perhaps the most well-recognized white-spotted breed, many other breeds also have prominent white markings. We therefore investigated whether the *KIT* noncoding deletion segregated in other breeds, and how variant status might align with patterning characteristics defining these breeds. Here, we mapped 548 publicly available genomes representing 13 breeds to our bespoke reference genome (Materials and Methods, fig. S4, and table S3). Notably, all characteristically spotted breeds appeared to be fixed, or near fixed, for the deletion allele (frequencies ranging from 0.97 to 1). The reverse was true in the most nonspotted breeds, although the deletion did segregate in Jerseys, Red Angus, Gelbvieh, and *Bos indicus* cattle (frequencies ranging from 0.03 to 0.33; table S3). The intermediate-length allele was observed only in characteristically nonspotted breeds (Limousin, Jersey, Red Angus, Angus, and Gelbvieh) and was the minor allele in all cases (frequencies ranging from 0.08 to 0.25).

Since the relationship between variant status and white markings might be due to shared ancestry more generally, we performed phylogenetic analyses of animals within the same multibreed dataset. When considering only a 10-kbp region centered on the *KIT* upstream deletion, spotted breeds clustered together (Fig. 1D), but these relationships could not be discerned when considering chromosome-wide sequence identity (fig. S5). These findings support the hypothesis that the *KIT* deletion, or a closely related haplotype thereof, also contributes to spotting in these breeds.

### Epistasis at the *KIT* locus

**Fig. 2.**
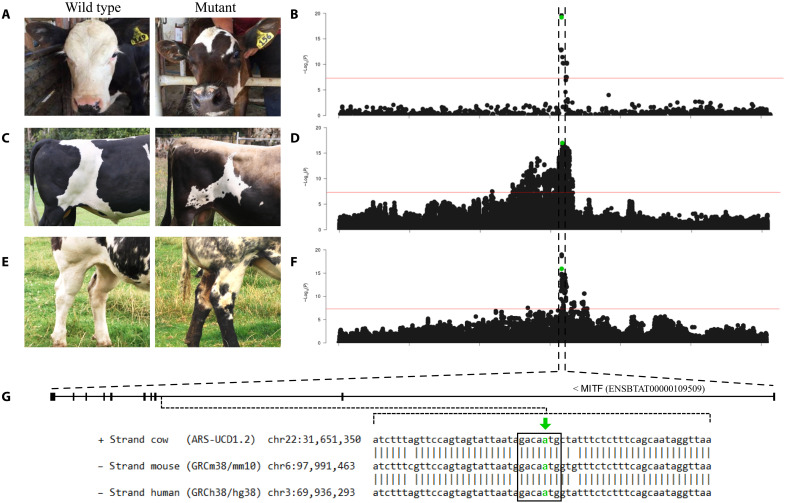
Epistatic interactions between structural variants at the *KIT* locus and the *MITF* Chr22 g.31651379A>G regulatory variant. (**A**) Image of white-faced (wild type; left) and splotchy-faced (mutant; right) calves and (**B**) Manhattan plot based on association results from chromosome 22 for this trait, with the top variant mapping to Chr22 g.31651404T>C, *P* = 2.41 × 10^−20^. (**C**) Zoomed in image of a “clean” white coat spot (wild type; left) and speckled spot (mutant; right) and (**D**) Manhattan plot from chromosome 22 for this trait, with the top variant mapping to Chr22 g.31651379A>G, *P* = 1.04 × 10^−17^. (**E**) Images showing the “black socks” trait, with a wild-type animal shown at left, and a black socks case at the right. (**F**) Manhattan plot from chromosome 22 for this trait, with the top variant mapping to Chr22 g.31650821T>A, 1.11 × 10^−19^. (**G**) *MITF* gene structure and multispecies alignment highlighting the highly conserved SOX10 binding site (outlined in black), with the candidate causative variant (Chr22 g.31651379A>G) highlighted in green [here and in Manhattan plots; (B), (D), and (F)].

Several other breed-defining coat characteristics have been mapped to the *KIT* locus in cattle, including the white-face trait in Herefords (*6*, *16*). While typically considered a dominant trait, we were aware of cases of incomplete penetrance in crosses between Herefords and some Jersey and Angus cattle, with calves presenting “splotchy” colored faces (Fig. 2A). Since the *KIT* deletion might be expected to interact with the *KIT* serial duplication underlying the white-face trait (*6*, *16*), we conducted an association analysis of 128 Hereford-cross calves that presented a mixture of splotchy and pure white faces (table S5). Genotype data were derived from a custom SNP chip that contained probes for the *KIT* deletion variant (table S6), and all calves were found to carry at least one copy of the deletion allele (as expected given that Hereford cattle are near fixed for the variant). Unexpectedly, neither the *KIT* deletion nor any other *KIT* locus variants appeared to be associated with the splotchy face trait (smallest *P* = 0.005 within a 20-Mbp interval centered on the deletion; fig. S6A). However, a strong signal was detected on chromosome 22 for splotchy face status (Fig. 2B), where the lead variant was in strong linkage disequilibrium (LD; *R*^2^ = 0.94) with the *MITF* candidate variant implicated in our previous GWAS of white spotting [smallest *P* = 2.41 × 10^−20^ for Chr22 g.31651404T>C rs208958980 versus *P* = 5.97 × 10^−20^ for Chr22 g.31651379A>G rs209784468 (*9*)].

Having highlighted this epistatic relationship, we wondered what other patterning interactions might be apparent in breeds differentially segregating for these loci. Upon reevaluation of phenotypes within our white spotting dataset, we noted that some animals, particularly those homozygous for the *MITF* SNP, presented with black speckles within patches of white on the coat (Fig. 2C). We were also aware of a unique leg pattern observed in some Belgian Blue animals, a breed that typically presents white legs although may also display mottled black “socks” (Fig. 2E). To assess the potential role of the *KIT* and *MITF* regulatory variants in these contexts, we performed GWAS in both cases. For the speckly trait, we scored 242 white-spotted Jersey and Holstein- Friesian × Jersey crossbred bulls (table S7) and assessed 135 Belgian-Blue animals segregating for the black socks phenotype (treating each as categorical variables). These analyses revealed strong associations in both cases, with both signals comprising single QTL locating to the *MITF* gene (Fig. 2, D and F). In the case of the speckly trait analysis, the candidate causative *MITF* SNP (Chr22 g.31651379A>G) was the most significant variant genome-wide (*P* = 1.04 × 10^−17^). This same variant also ranked near the top of the association peak for the black socks trait (Fig. 2F; *P* = 1.15 × 10^−16^ for Chr22 g.31651379A>G versus *P* = 1.11 × 10^−19^ for Chr22 g.31650821T>A rs210634530), suggesting the *MITF* noncoding SNP as likely responsible for all epistatic effects.

Comparing effects across all traits, it is noteworthy that the chr22:31651379 bp “G” allele associated with splotchy face, black speckles, and black socks is the same allele that decreases white spotting percentage. Curiously, however, this “pigment increasing” allele appears to be the derived versus ancestral form, based on the presence of the Chr22 g.31651379 “A” allele in both human (GRCh38/hg38) and mouse (GCRm38/mm10) genomes. Since pigmented hair assumedly represents the wild-type state, this suggests a gain of function mechanism to the chr22:31651379 bp G allele and possible coselection of the variant in conjunction with other color/patterning loci. Alternatively, selection of this “pigment-increasing” allele might be due to some pleiotropic impact on one or more other animal performance traits. To test this hypothesis, we evaluated the effect of the *MITF* SNP on 23 other traits assessed as part of dairy animal selection activities (*N* > 38,000 to 73,000 animals per trait; Materials and Methods). While some significant associations were apparent (*P* < 0.05; table S8), these effects were small and would seem unlikely to have elicited historical selection of the allele. Testing the *KIT* structural variant tag SNP (rs463810013) using the same approach likewise yielded no compelling effects for that variant (table S8).

### Functional analysis of the *KIT* and *MITF* white spotting variants

To functionally assess the regulatory potential of the candidate white spotting variants at the *KIT* and *MITF* loci, we performed editing experiments in both cells and mice. First, we identified mouse sequence homologous to the 6.9-kbp cow ancestral alleles and used clustered regularly interspaced short palindromic repeats (CRISPR)–Cas9 to knockout two fragments of this sequence in mouse melanoblasts [melbA cells; (*17*)]. These lines represented heterozygous knockouts encompassing a larger conserved sequence (~4.1 kbp deleted) and a ~1.3-kbp subsequence containing the MITF binding sites of interest (Materials and Methods and figs. S2C and S7A). A reduction in *Kit* expression was observed between full-length knockout lines and controls, although no significant differences were observed in lines bearing the shorter-length edit (fig. S7A). Likewise, cell cycle analysis via fluorescence-activated cell sorting suggested slower cell cycle progression in lines with full-length knockouts, consistent with a reduction in *Kit* activation and downstream signaling (fig. S7B) (*18*). No effect was apparent for short-length knockout lines, and wound healing assays conducted to assess cell migratory potential showed no differences between any of the edited genotypes (fig. S7C).

**Fig. 3.**
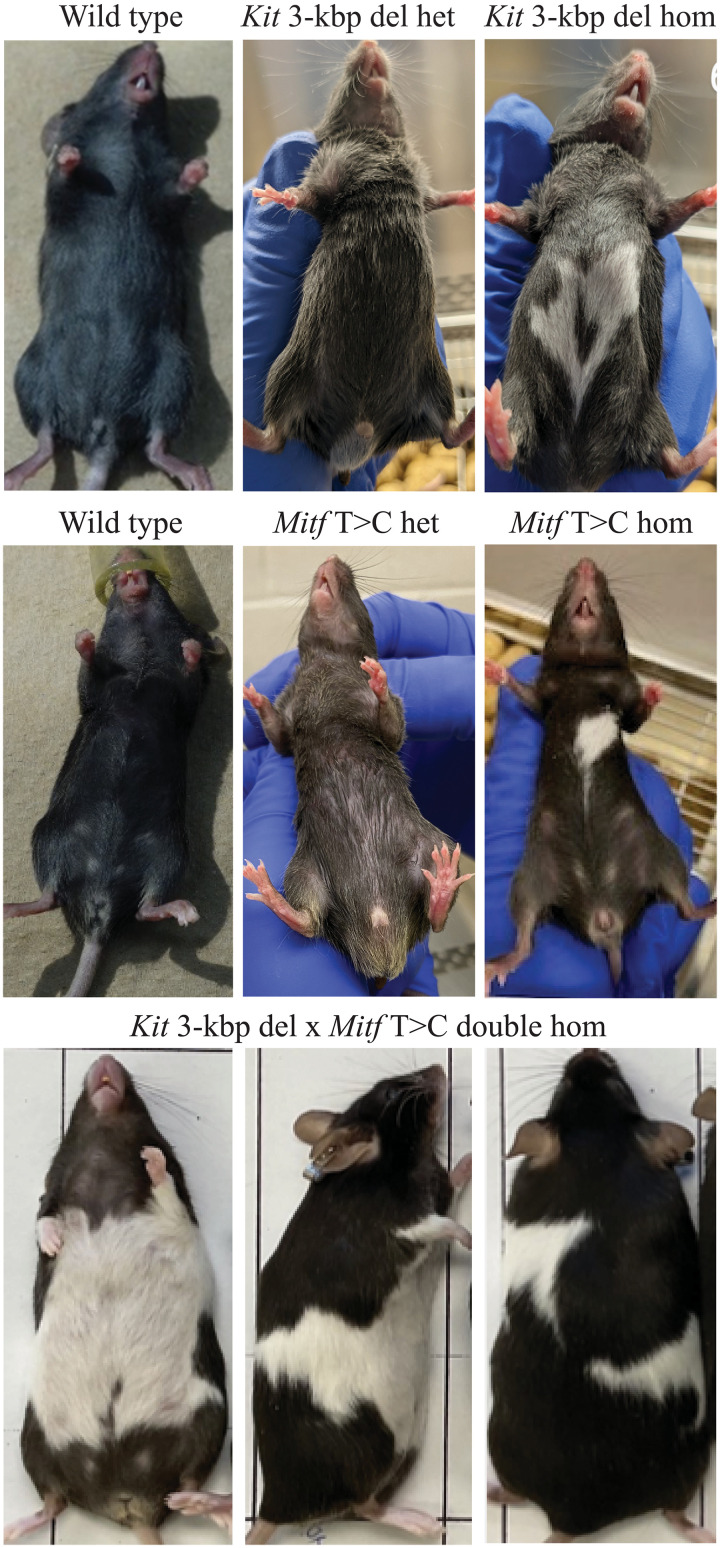
White spotting phenotypes observed in C58BL/6 mice edited for variants modelling the cattle *KIT* deletion allele (*Kit* 3-kbp del) and *MITF* Chr22 g.31651379A>G (*Mitf* T>C) variant. Wild-type, heterozygous, and mutant mice for the *Kit* variant are shown in the top three panels. Heterozygotes showed either solid coats (*N* = 19) or a small number of white belly hairs (*N* = 10; mouse shown). All homozygous mutants showed prominent white belly spots (*N* = 7). *Mitf* T>C model mice are shown at the middle. Heterozygotes presenting mostly solid coats (*N* = 14; mouse shown) or a small white belly spot (*N* = 1), with the two *Mitf* T>C homozygotes both showing a single white belly spot. The bottom shows ventral, lateral, and dorsal views of a double homozygous mouse bred through intercrossing heterozygous *Kit* 3kbp del and *Mitf* T>C lines. Double homozygotes (*N* = 2) had similar, near complete depigmentation of the belly that extended to lateral band patterns.

Since melanoblast colonization in vivo is particularly sensitive to changes in proliferation caused by mutation of *Kit* (*19*), we next generated a mouse model of the variant. Here, CRISPR-Cas9 was used to excise 3.0 kbp between chr5:75,488,479-75,491,494 bp in a C58BL/6 mouse background (genome build GRCm38/mm10; Materials and Methods and fig. S2C). While the cell-based results demonstrated relatively modest changes between genotypes, edited mice strongly supported the causality of the *KIT* noncoding deletion, where heterozygotes presented a small white belly spot or solid-colored coats, and all homozygous mice had large white belly spots (Fig. 3).

We also sought to test the function of the *MITF* intronic SNP (Chr22 g.31651379A>G) in mice. This was acknowledging the role of the mutant allele as a likely gain-of-function, pigment-increasing variant—although we reasoned that patterning effects might be apparent regardless. Here, we performed precise editing of the conserved base (Chr6 g.97968472T>C in mice), and edited mice showed phenotypes similar to *Kit* deletion mice. Heterozygotes showed variable expressivity with either small white belly spots or solid black coats, with homozygous mice showing white belly streaks (Fig. 3). Several lines with small off-target noncoding indels adjacent the target *MITF* SNP showed notable splashed white coat patterns (fig. S8). Double homozygotes generated by intercrossing the *KIT* deletion mice with *MITF* SNP carriers likewise resulted in extensive depigmentation reminiscent of Holstein-like coat patterns (Fig. 3). These observations highlight the functional importance of the intronic *MITF* sequence affected by the Chr22 g.31651379A>G bovine SNP, where the perfectly conserved murine sequence has been previously shown to mediate *Mitf* transcriptional activation via Sox10 binding (*20*). These results also confirmed the likely causality of this SNP, although they highlight a mechanistic contrast between species—given that the depigmentation of edited mice is essentially opposite to the pigmentation enhancement effects seen in cattle.

## DISCUSSION

Here, we present a detailed genetic analysis of the two largest-effect QTL underlying the hallmark white spotting of HF cattle. These two QTL map to the *KIT* and *MITF* loci on chromosomes 6 and 22, respectively—effects that have been previously reported in genetic mapping studies of animals with HF ancestry (*8*, *9*, *21*). In the most recent of these studies (*9*), we suggested a possible causative variant for the *MITF* QTL, although no such variant could be identified for the chromosome 6 location. In the current study, we provide compelling evidence supporting the causality of a ~6-kbp structural variant underlying this effect. This deleted segment is highly conserved, and the equivalent human sequence encompasses an Encyclopedia of DNA Elements (ENCODE)-annotated candidate cis-regulatory element (*13*). This sequence also contains a large number of ChIP-seq–derived transcription factor binding sites including those for *MITF* (*14*). Prior identification of the variant has likely been confounded by its status as a structural variant and its mechanism as a long-distance–acting regulatory sequence (operating ~114 kbp upstream of *KIT*). The variant also emphasizes how the composition of the reference assembly can complicate discovery, given that the Hereford-based reference genome integrates the deleted, mutant allele.

The finding that Hereford and HF cattle are both near-fixed for the white-increasing *KIT* allele was somewhat unexpected—given that the coat spotting of these breeds is phenotypically distinct. Aside from the obvious difference in face pigmentation of Herefords, coat depigmentation is concentrated down the spine and undercarriage of the animal, as opposed to the more generally pied coats of HFs. However, recent analysis of the white-face trait in Herefords and Simmentals suggests a segmental duplication upstream of *KIT* as likely responsible (*6*), so haplotypes of this variant and the deletion allele reported here might be expected to generate unique patterns across breeds. This diversity of patterning is also seen in other breeds found to be fixed for the *KIT* deletion allele—for example, Normande that are more speckled in appearance. Both cis and trans interactions may be quite common for patterning traits, as evidenced by the epistatic effects detailed in the current study. We observed three distinct examples of unique pattern modifications linked to the *MITF* intronic SNP, including reversion of the Hereford white-face, speckling of white coat spots, and repigmentation of the typically white legs of Belgian Blue animals. This is the same *MITF* variant we previously attributed to the chromosome 22 white spotting QTL, and as expected, the allele that increases white coat spotting (*9*) is the same allele that enables full expression of the white-face, white legs, and solid white spots free of intermingled speckles (*9*). What was unexpected, however, is that this “white increasing” allele appears to be the ancestral versus mutant form*.* Given that ancestral *Bos taurus* animals might be assumed to be solid colored, this finding raises the question of how a “gain of pigmenting function” allele would be recognized and selected. One possibility is that this allele has pleiotropic impacts on other phenotypes, although we noted no such compelling effects in analyses of 23 other animal performance traits. An alternative (and we think more plausible) possibility is that historical animal breeders selected the variant precisely because of its epistatic modification of other interesting coat characters.

Our cellular experiments to model the effects of the *KIT* upstream structural variant showed moderate impacts on *Kit* gene expression and melanoblast proliferation. By comparison, a mouse model of the variant unambiguously demonstrated the regulatory activity of this sequence but with variable expressivity in the observed phenotypes. This variation is consistent with melanoblast colonization being driven in part by stochastic variation in cell behavior (*19*), which may similarly contribute to the coat variation observed in individual cattle. The *KIT* variant described here is in addition to several other *KIT* expression variants that underlie notable coat patterns in cattle, namely, the white face variant mentioned above, color-sided patterns of Belgian Blue, White Park, and Gloucester animals, and the “line-back spotting” of Pinzgauer cattle (*4*, *5*, *16*, *22*, *23*). Together, these variants highlight the regulatory diversity of the *KIT* gene, creating unique phenotypes presumably through control elements with different functional, spatial, and temporal roles during melanoblast development.

Our *MITF* mouse model likewise confirmed the regulatory capacity of the bovine variant. This SNP overlaps a conserved and functionally validated SOX10 binding site in the melanocyte-specific promoter of *MITF*, a motif that works synergistically with an adjacent PAX3 binding site to activate *MITF* expression (*20*). Curiously, however, the depigmentation effects are the reverse of what might be expected from observations in bovine. In cattle, the derived allele is in association with increased pigmentation in areas that are depigmented by virtue of other patterning variants (for example, the *KIT* white face serial duplication or the white spotting structural allele reported here). The mechanism of this contrast is unknown, although it may reflect species-specific differences in melanocyte subpopulations. In mice, epidermal melanocytes in the trunk and limbs are lost shortly after birth—restricting the developmental window in which patterns can form (*24*)—whereas they are retained in humans and cattle (*25*). In addition, in both mouse and chick, melanocytes arise from two distinct sources: an early neural crest-derived population and a later Schwann cell precursor (SCP)—derived population (*26*). The relative contribution of these populations to pigmentation patterns remains poorly understood across species. In mice, SCP-derived melanocytes contribute more prominently to distal regions such as the paws (*27*). Therefore, in cattle—where epidermal melanocyte populations are maintained—a variant that affects SCP-derived melanocytes specifically may have a strong effect on distal regions and promote the generation of discrete pigment patches within otherwise unpigmented areas of the epidermis.

In conclusion, we report the detailed characterization of the two major white spotting loci in HF cattle, highlighting long-range and intronic cis-regulatory variants in the *KIT* and *MITF* genes. Multibreed analyses suggest that the variants affect coat phenotypes across a variety of breeds and produce distinct patterning effects through interactions with other major loci. Future cross-breeding experiments involving additional breeds will help better characterize these effects and may assist breeding of animals with other unusual and desirable coat patterns.

## MATERIALS AND METHODS

### Cattle populations and ethical approvals

The cattle populations described in this paper represented several cohorts. Table S9 summarizes breed and cohort information and the respective analyses performed in this study. Briefly, a cohort of previously described sequenced cattle (*N* = 565) was used as the initial discovery dataset (*28*), and sequence data for an additional 562 cattle were later incorporated and used as an imputation reference dataset (total *N* = 1126 whole genome-sequenced dairy cattle). These animals consisted of commercially farmed, purebred HF (*N* = 116), purebred Jersey (J; *N* = 95), or crossbred HF × J cattle (*N* = 354) of both sexes, where “purebred” animals were defined on the basis of a breed proportion of ^16^/_16_ from a four-generation pedigree. Blood samples from three HF bulls, three J bulls, and two Hereford bulls were used to derive DNA for PCR and long-read sequencing. Genotype and phenotype data were available for 2976 cattle (*9*), where that population had an overlap of 499 animals with the initial genome-sequenced discovery dataset. DNA sequence data from Angus, Red Angus, Charolais, Limousin, Maine-Anjou, Montbeliarde, Normande, Gelbvieh, *B. indicus*, Hereford, and Holstein cattle were downloaded in the form of fastq sequence files from the National Center for Biotechnology Information (NCBI) Sequence Read Achieve (*N* = 332; ERP010431, SRP017441, and SRP245473) and combined with purebred HF and J cattle sequence data from the discovery dataset for phylogenetic analyses. Genotype and phenotype data were available for white-faced and splotchy-faced Angus × Hereford (*N* = 21) and HF × J × Hereford (*N* = 107) calves, derived as described in the following sections. Speckling data were available for 242 bulls from the sequenced discovery dataset (J *N* = 37 and HF × J *N* = 205). Analysis of the black socks phenotype was based on analysis of 135 Belgian Blue animals.

All large animal experiments were conducted in strict accordance with the rules and guidelines outlined in the New Zealand Animal Welfare Act 1999. Approval was sought from the AgResearch Animal Ethics Committee, Hamilton, New Zealand for sampling and trait scoring of the Hereford cross calves used in this study (approval AEC 15236). The scoring procedures for white spotting that were not based on preexisting photographs were approved by the AgResearch Animal Ethics Committee (approval AEC 14090). All other cattle data were generated as part of routine commercial activities that are outside the scope of those requiring formal committee assessment or ethical approval (as defined by the above guidelines). Mouse models were generated and bred under McGill University animal use protocols (AUP 4437 and 7843).

### Whole-genome sequence and genotype data

Whole-genome sequencing and read-mapping were performed on a population of 1126 cattle as previously described (*28*, *29*). Briefly, DNA samples for all cattle were sequenced using 100- or 150-bp paired-end reads on the Illumina HiSeq 2000 platform. Read mapping was initially performed on the previously published cohort of 565 cattle (*30*) using the ARS-UCD2.1 genome build (*31*) and BWA MEM (v0.7.17) software (*32*). An additional 562 cattle were also sequenced after the discovery of the candidate structural variant. Sequence data representing this combined dataset (*N* = 1126 cattle) were mapped to our bespoke reference genome (described in the “Creation of a structural variant-augmented reference genome” section below) using BWA MEM. The mean mapped read depth across chromosome 6 for this dataset was 15×. The methods outlined above were also used to map publicly available sequence data from an additional 332 cattle representing a variety of spotted and nonspotted cattle breeds (table S9), resulting in a mean mapped read depth of 13× for that dataset.

Microarray-based genotype data were available for the cattle used in the white spotting association analysis (*N* = 2477). These data were generated by GeneSeek (Lincoln, NE, USA), using a variety of platforms, including the Geneseek GGPv1, GGPv2, GGPv3, GGP50k, Illumina BovineSNP50, or BovineHD 777k SNP chips, as previously described by Jivanji *et al.* (*9*). Step-wise imputation to sequence resolution genotypes was performed using Beagle 5.0 software (*33*) and has been described by Reynolds *et al.* (*34*). Variants were subsequently filtered for imputation quality (variants with a dosage *R*^2^ < 0.7 were removed) and rare variants (variants with a homozygous alternative count ≤ 5 were removed) to avoid potentially spurious associations.

Tissue samples were obtained from ear tissue biopsies from 128 white-faced or splotchy-faced Angus × Hereford and HF × J × Hereford calves, and DNA extraction was conducted at GeneMark (Hamilton, New Zealand), using the Qiagen BioSprint kit. Genotyping was conducted using the GeneMark research version 1 (RESv1) SNP chip, which contained nine custom probes designed to genotype the *KIT* structural variant (table S6).

### Identification and genotyping of a candidate structural variant at the *KIT* locus

As part of our previous analyses, we reported a candidate structural variant that mapped downstream of the *KIT* gene to Chr6:72,060,120-72,060,450 bp on the UMD3.1 bovine reference build (*9*). Paired-end short-read sequence data from 565 HF, J, and crossbred cattle were mapped to the ARS-UCD1.2 reference genome (*5*) using BWA MEM (v0.7.17) (*32*), and this region was investigated using the Integrative Genomics Viewer (IGV) software (*35*). The alignments were visualized by insert size, pair orientation, and soft clip status in IGV.

The Unix grep tool was used to genotype cattle for mutant and wild-type sequences observed between Chr6:70,052,523-70,052,965 bp and Chr6:70,369,307-70,396,749 bp, either side of the *KIT* gene. Twenty-nucleotide search strings that encompassed 10 bp from the soft-clipped region and 10 bp from the reference consensus region (table S10) from both candidate sites were used to query raw fastq sequence files. These analyses were restricted to cattle samples that had a minimum average read depth of 10× coverage (*N* = 152 cattle). A search using the Basic Logical Alignment Search Tool [BLAST; (*36*)] against the ARS-UCD1.2 bovine reference genome confirmed each search string to be unique, with no sequence homology with any other region of the genome identified. The search strings that incorporated the soft-clipped reads were considered representative of the mutation breakpoints, and the corresponding 20 bp ARS-UCD1.2 sequence string represented the reference form of these sites. Reverse complement sequence for each of these search strings was also queried. The proportion of alternate search string matches observed at any one site (the number of alternate form matches/total matches detected) was used as a proxy for genotype at the upstream and downstream candidate sites. The correlation between the proportion of alternate search string matches observed at any one site and the genotype at rs463810013 was computed using the dplyr (v0.7.8) package in R (*37*).

### DNA extraction

To characterize candidate structural variant junctions, 10-ml blood samples were obtained from three J bulls, three HF bulls, and two Hereford bulls, selected on the basis of their genotypes at previously identified white spotting-associated tag SNP rs463810013 (table S2) (*9*). To extract the highest molecular weight DNA possible, our DNA extraction protocol embedded mononuclear cells in an agarose matrix before cell lysis, aiming to prevent excessive shearing of the DNA and permit efficient long-range PCR. Samples were collected in heparin tubes and processed on the same day. Briefly, peripheral blood mononuclear cells were isolated from whole blood by a series of red blood cell lysis and centrifugation cycles. Pelleted white blood cells were gently resuspended in an appropriate volume of phosphate-buffered saline (PBS) to give a final concentration of 2 × 10^7^ cells/ml and warmed to 37°C. The cell suspension for each sample was gently mixed with 2% low melting agarose in PBS in equal volumes and cast into 100-μl moulds. Once the agarose cell suspension plugs had solidified, samples were halved and incubated in lysis solution at 50°C for 48 hours. The 50-μl agarose plugs were washed three times with wash buffer and stored in wash buffer at 4°C for several days until required. Each 50-μl agarose plug was estimated to have had 1 × 10^6^ cells before cell lysis, equating to ~6600 ng of DNA per plug.

Genomic DNA was extracted from the 50-μl agarose plugs using the NucleoSpin Gel and PCR Clean-up kit (Machery Nagel). Briefly, agarose plugs were placed into a clean tube with 200 μl of Binding Buffer NTI buffer. The plugs were incubated for 5 to 10 min at 50°C and gently mixed every 2 to 3 min until the gel slice was completely dissolved. The sample was then loaded into a NucleoSpin Gel and PCR Clean-up column and centrifuged at 11,000*g* for 30 s. The flowthrough was discarded, and the column was washed with 500 μl of wash buffer NT3 buffer. Samples were centrifuged for 30 s at 11,000*g*, the flowthrough was discarded, and this step was repeated. The silica membrane was dried by being placed into a clean collection tube, centrifuged for 1 min at 11,000*g*, and then incubated at 70°C for 2 to 3 min to remove excess ethanol. The DNA was eluted using 15 μl of water warmed to 70°C. The sample was centrifuged for 1 min at 11,000*g*, and this step was repeated before the final DNA sample was obtained.

### Long-range PCR and minion sequencing of candidate structural variant sites

Primer pairs were designed to amplify a 6337-bp region at the upstream candidate site (Chr6:70,048,910-70,055,246 bp) and a 4749-bp region at the downstream candidate site (Chr6:70,394,382-70,399,130 bp; table S10), selected to target “cleanly aligned” sequences based on visualization of genome sequence alignments. A touch-down PCR was conducted using the KAPA LongRange PCR kit (Kapa Biosystems). The initial denaturation step was conducted at 95°C for 30 s, followed by 10 cycles; denaturation at 95°C for 30 s, annealing at 70°C for 30 s (decreasing by 1°C per cycle); extension at 68°C for 13 min for the upstream site and 72°C for 4 min for the downstream site, followed by an additional 25 cycles with annealing at 60°C. The PCR products were loaded and run on a 1% agarose gel for 60 min at 100 V to estimate the amplicon size.

The PCR amplicons were purified using AMPure XP beads (Beckman Coulter), barcoded to enable pooling of samples, and then used to construct a sequencing library using the EXP-NBD103 and SQL-LSK109 kit (Oxford Nanopore Technologies) as per the manufacturer’s instructions. The first library, targeting the upstream candidate site, was constructed using DNA from across the eight samples, loaded onto a FLO-MIN106 flow-cell (Oxford Nanopore Technologies). Amplicons representing the upstream candidate site were sequenced for 2 hours. The second library, targeting the downstream candidate site, was constructed using DNA from across the same eight, loaded onto a FLO-MIN106 flow cell, and sequenced for 40 min. The sequencing depth per sample can be found in table S2.

### Creation of a structural variant-augmented reference genome

Sequence reads from the minion sequencer were base-called using Guppy basecaller (v4.0.14) (*38*), with the samples then separated on the basis of their barcodes by Guppy barcoder (v4.0.14), and subsequently aligned to the ARS-UCD1.2 reference genome using minimap2 (v2.14) (*39*). A consensus sequence was added to the ARS-UCD1.2 reference genome as an alternative contig. Short-read sequence data from two cattle previously identified to have the structural variant using the grep-based method (see the “Identification and genotyping of a candidate structural variant at the *KIT* locus” section) and two cattle that had the deleted allele were aligned to the modified reference genome using BWA MEM (v0.7.17) (*32*). The sequence reads aligned to the amplicon-derived consensus were manually inspected in IGV (*35*), and obvious, easy-to-resolve errors in the consensus sequence were manually corrected.

The final consensus sequence representing the ~13-kbp amplicon generated from amplification of the upstream candidate site was searched against the ARS-UCD1.2 reference genome using BLAST (*36*) to identify the likely insertion site. The ARS-UCD1.2 chromosome 6 reference sequence was split at the candidate insertion point using SAMtools (*40*), and the structural variant sequence was inserted ~114 kb upstream of the *KIT* gene (between Chr6:70,052,697 bp and Chr6:70,052,698 bp). Notably, the inserted sequence was flanked by blocks of sequence 42 bp long, identical except for one base pair (TGAACTTCCTGATGTT[G>C]AAGCTGGTTTTAGAAAAGGCAGA). The G allele variant of the 42-bp sequence mapped to the 3′ end of the ancestral allele and appeared to be a fragment of a bovine retrotransposable element (RTE-BovB) long interspersed nuclear element. A BLAST of 42-bp sequence revealed that it was observed 1905 times across the ARS-UCD1.2 reference genome, including at the previous downstream candidate site, mapping to Chr6:70,396,679-70,396,718 bp. Short-read sequence data from the four cattle previously used to correct errors in the consensus sequence were used to confirm the insertion site. Sequence alignments were visually inspected in IGV (*35*), adjusted, and remapped until soft-clipped reads were no longer observed across the breakpoints. All minION sequence data were also remapped to the bespoke reference genome using minimap2 (*39*), and the structural variant was visualized in IGV. A colocating 850-bp deletion was observed in the J2 sample, mapping to Chr6:70,054,845-70,055,695 bp in the bespoke reference. This haplotype also differed from the long-form ancestral allele by 71 other polymorphic variants.

### Genotyping the *KIT* structural variant

Sequence alignments representing HF (*N* = 280), J (*N* = 188), and HF × J (*N* = 659) cattle and 332 other spotted and nonspotted breeds mapped to the bespoke reference genome were used to genotype the *KIT* structural variant. CNVnator (v0.3.3) (*41*) was used to predict the presence of the deletion allele and intermediate-form ancestral allele based on average read depth in these regions within their sequence context. The CNVnator-predicted copy number calls across the deletion and intermediate-form ancestral alleles were confirmed or adjusted on the basis of visual inspection of plots generated by Samplot (*42*) that summarized read-depth and split read information across the structural variant site. If deletion status remained ambiguous, sequence reads were manually inspected in IGV (*35*) to confirm the structural variant genotype.

### Variant calling and imputation

Genotype data for association and phylogenetic analyses were called from sequence data aligned to the bespoke chromosome 6 reference using the Genome Analysis Toolkit (GATK) HaplotypeCaller (v4.1.8.1) (*43*). This step used the same alignments described above, representing 548 *B. indicus*, Angus, Red Angus, Charolais, Limousin, Maine-Anjou, Montbeliarde, Normande, Gelbvieh, Holstein, Hereford, NZ HF, and NZ J cattle. To capture genotypes that collocated to the structural variant, we used default parameters to variant call all cattle with homozygous genotypes across the *KIT* structural variant (regardless of allele). Sequence alignments for the remaining cattle that were either hemizygous for the deletion allele or intermediate-form ancestral allele were split at the allele-specific deletion junctions using SAMtools (*40*) so that these regions could be interrogated separately. Variant calling across the sequence representing a hemizygous deletion state was conducted using HaplotypeCaller (v4.1.8.1) software (*43*) with ploidy set to one. The remaining segments of sequence aligned to the bespoke reference were variant called using default parameters, and the resulting variant called files were concatenated using BCFtools (*40*).

For the purpose of downstream imputation and association analyses, the *KIT* variant genotype (as established by methods described in the “Genotyping the *KIT* structural variant” section) was summarized as one representative triallelic variant for 280 HF, 188 J, and 659 crossbred (i.e., the imputation reference dataset; see table S9). The GATK HaplotypeCaller (v4.1.8.1) software (*43*) was used for variant calling on this sequenced dataset mapped to the ARS-UCD1.2 reference genome, and the *KIT* structural variant genotype was manually added to the variant call format file at the nonvariant Chr6:70,057,008 bp site. This sequenced cohort (*N* = 1126) was used as a reference dataset to impute the triallelic variant into the phenotyped population (i.e., animals with white spotting data). The reference dataset was phased using Beagle 5.1 (*33*), and imputation was conducted across Chr6:60-80 Mbp. The intermediate-form ancestral allele was imputed into the phenotyped population with an allelic *R*^2^ of 1, and the deletion allele was imputed with an allelic *R*^2^ of 0.99.

### Phenotypes, population structure adjustments, and association analyses

White spotting phenotype data were available for 2967 NZ dairy cattle (*9*). Genotypes were used to categorize this population into four different overlapping datasets to test the association between alternative structural variant states and the proportion of white spotting. The first dataset included animals with the long-form ancestral or deletion allele (*N* = 2596), the second dataset included animals with the long-form or intermediate-form ancestral allele (*N* = 85), and the third dataset included animals with the intermediate-form ancestral or deletion allele (*N* = 2507). The last dataset combined animals that had either the long-form or intermediate-form ancestral allele for contrast with those that had the deletion allele (*N* = 2967). Genomic relationship matrices (GRMs) were generated using Genome-wide Complex Trait Analysis software (GCTA; v1.93.2beta) (*44*) to address population stratification due to breed and relatedness in the association models. These GRMs were calculated on the basis of a subset of 19,354 markers from the Illumina Bovine SNP50 platform, having been filtered on the basis of minor allele frequency (those with a minor allele frequency < 0.02 were removed), deviation from Hardy Weinberg equilibrium (those with a *P* < 0.15 were removed), missing genotype rates (those with a genotyping rate < 0.01 were removed), and high LD with another marker on the panel (those with pairwise *R*^2^ > 0.9 were removed). To avoid fitting variants that were in LD with the *KIT* structural variant, markers from chromosome 6 were also excluded. Association analyses were performed on 152,072 sequence-resolution markers mapping between Chr:60-80 Mbp. The GCTA software (*44*) was used to conduct mixed linear model–based association analyses (MLMA), which incorporated the GRM described above.

Face color (white or splotchy) was reported by farmers, or scored using photographs taken by farmers, on 21 Angus × Hereford calves and 107 HF × J × Hereford calves (tables S5 and S8). The speckling phenotype was categorized on the basis of the presence or absence of black speckles in areas of white on the coat, scored from photographs of 37 J and 205 J × HF bulls. For both traits, a “leave one chromosome out” (LOSO) approach was used to calculate 29 GRMs, where each GRM lacked one autosome to avoid double fitting when testing the effect of candidate variants on that excluded autosome, as previously described by Jivanji *et al.* (*9*). The GRMs were calculated using variants from the RESv1 platform used to genotype these cattle (*N* = 21,159 variants), with quality filtering applied as described above for the proportion of white spotting analyses. The GCTA software was used to perform MLMA using the RESv1 resolution genotype data for face color and whole-genome sequence resolution genotypes for speckling.

For analysis of the black socks phenotype, we leveraged a dataset of 135 Belgian Blue animals with incompletely white coats. Visual inspection identified 56 black socks cases, and all animals were genotyped using a custom 20K SNP array (EuroGenomics v9 SNP array, Illumina, Inc.). Genotype data were augmented via imputation in two steps: first to high-density using 890 Belgian Blue animals genotyped with the BovineHD BeadChip as a reference (~770,000 variants) and then to whole-genome resolution using sequenced Belgian Blue animals as a reference (*45*). Phasing was performed using Shapit4, followed by imputation with Minimac4. For association testing, we used the same LOSO approach to that applied for analysis of the splotchy and speckly traits. Genotype data from the custom 20k SNP array were used for the construction of GRMs and were first filtered on the basis of minor allele frequency (< 0.02), genotype missingness (genotyping rate < 0.8), and high LD (pairwise *R*^2^ > 0.9). GCTA software was also used for analysis of the black socks trait, incorporating the GRMs described above and using sequencing-resolution variants (*N* = 10,366,189).

We used a *P* value of 5 × 10^−8^ as the significance threshold to account for multiple hypothesis testing for GWAS. To assess whether candidate variants of interest wholly explained association signals for the white spotting trait, variant genotypes were fitted as fixed effects as part of subsequent analyses.

Pointwise association statistics for the *KIT* and *MITF* patterning–associated variants were also derived for 23 animal performance traits. The chr6:70072417C>T rs463810013 tag SNP was assessed as a proxy of the *KIT* structural variant, and the assumed causal chr22 g.31651379A>G rs209784468 variant was assessed directly (although both representing imputed genotype data). Association effects for milk production traits were obtained from a previous study (*46*). Phenotypes were preadjusted for parity, stage of lactation, herd by test day, breed, and heterosis effects, and the additive effect of each SNP was estimated using mixed model association statistics from an infinitesimal model using the Bolt-LMM software (*47*). Variant effects for all remaining animal performance traits were estimated using pedigree-based models in ASReml-R (*48*), using phenotypes from our previous study (*28*). Each SNP was fitted as a quantitative variable in a separate model, with covariates included for the proportions of NZ HF ancestry, US HF ancestry, Jersey ancestry, and heterosis effects. Pedigree relationships were accounted for by fitting a pedigree-based relationship matrix.

### Phylogenetic analyses

Consensus sequences for chromosome 6 were generated for 13 spotted and nonspotted breeds (see table S3) mapped to the bespoke reference genome using BCFtools consensus (*40*). A 10-kbp region encompassing the *KIT* structural variant (Chr6:70,051,190-70,061,190 bp) was extracted from each consensus sequence file. An average of 99 polymorphic variants was observed across all breeds. The “mash sketch” function from Mash (v2.3) (*49*) was then used to convert each sequence into MinHash sketches with the default *k*-mer (*k* = 21) and sketch sizes (*s* = 1000). Pair-wise Mash distances were calculated using the “mash dist” function. A neighbor-joining tree was constructed from the distance matrix using QuickTree (v2.5) (*50*) and visualized in FigTree (v1.4.4). These methods were then applied to the larger, whole chromosome 6 consensus sequences, which contained 797,267 polymorphic variants across breeds.

### *KIT* structural variant cell models

The mouse melanoblast cell line melbA (*16*) was cultured in RPMI 1640 media (21875-034; Invitrogen) supplemented with 10% (v/v) fetal calf serum, 40 pM Fibroblast Growth Factor 2 (FGF2), and mouse Stem Cell Factor (mSCF; 20 ng/ml). Cells were incubated at 37°C in humidified air containing 5% (v/v) CO_2_. Guide RNA (gRNA)–expressing constructs were prepared by annealing forward and reverse gRNA containing oligonucleotides and subsequent cloning into the BbsI site of pSpCas9(BB)-2A-GFP (PX458)—a gift from F. Zhang (Addgene plasmid no. 48138; http://n2t.net/addgene:48138; RRID:Addgene_48138; table S12). Cells were transfected with flanking gRNA pairs using the Neon (Thermo Fisher Scientific) electroporation system. Briefly, cells were grown to 90% confluence in a T75 flask, trypsinized, spun down, and resuspended at 250,000 cells in 20 μl of buffer R. One microgram of each gRNA flanking pair was added to each tube, and the cells were electroporated with two pulses of 1350 V for 20 ms. Cells were plated into optiMEM supplemented with 40 pM FGF2 and mSCF (20 ng/ml). Media was changed to growth media after 24 hours. Subsequently, the pooled cells were cloned by dilution into 96-well plates.

Primers flanking the gRNA binding sequences (table S12) were used to screen for deletion of the targeted sequences. The wild-type band was amplified using a touch-down protocol [10 cycles: denaturation at 98°C for 30 s; annealing at 70°C for 30 s (decreasing 1°C per cycle); extension at 72°C for 30 s, followed by an additional 20 cycles: denaturation at 98°C for 30 s; annealing at 60°C for 30 s; extension at 72°C for 30 s]. The large deletion was amplified using a touch-down protocol [10 cycles: denaturation at 98°C for 30 s; annealing at 72°C for 30 s (decreasing 1°C per cycle); extension at 72°C for 30 s, followed by an additional 20 cycles: denaturation at 98°C for 30 s; annealing at 62°C for 30 s; extension at 72°C for 30 s]. The small deletion was amplified by conventional PCR (35 cycles: denaturation at 98°C for 10 s; annealing at 67°C for 10 s; extension at 72°C for 5 s). All PCR reactions were performed with Phusion polymerase [New England Biolabs (NEB)] using GC buffer according to the manufacturer’s instructions. Clones were sequence verified using Sanger sequencing (Source Bioscience).

### Reverse transcription quantitative PCR of *KIT* structural variant cell model

Total RNA was extracted using the RNAeasy extraction kit (QIAGEN, Crawley, UK) following the manufacturer’s instructions, including a deoxyribonuclease (DNase) digestion with DNase I (QIAGEN, Crawley, UK). cDNA was synthesized using 1 μg of RNA, by reverse transcription using oligo dT18, RNase OUT, and Moloney Murine Leukemia Virus Reverse Transcriptase (M-MLV RT; Invitrogen, Thermo Fisher Scientific). Reverse transcription quantitative PCR was conducted with 1.5 μl of cDNA, 3 μl of distilled H_2_O free from RNase and DNase, 12.5 μl of PowerSYBR Green 2× master mix (Applied Biosystems, Warrington, UK), and 0.25 μM primers. Reactions were performed on a Bio-Rad CFX96 PCR system using the following parameters: 50°C for 2 min and 95°C for 10 min, followed by 40 cycles of 95°C for 15 s and 60°C for 1 min. Cycle threshold values were generated and compared to β-actin controls to determine gene expression using the CT(2^−ΔΔCT^) method (*51*).

### Wound healing assay

Cells were counted with a hemocytometer and plated on glass-bottom 24-well plates at 25,000 cells per well for 48 hours before a straight scratch was made down the center of each well using a 200-μl tip, followed by a media change. Cells were time-lapse imaged every 25 min overnight using a Zeiss LSM880 confocal microscope system with a 10× objective lens. Wound closure was quantified using ImageJ software (National Institutes of Health).

### Cell cycle analysis

Cells were counted with a hemocytometer and plated in six-well plates at 60,000 cells per well and allowed 24 hours to proliferate before trypsinization and fixation in 1 ml of ice-cold 70% ethanol for 16 hours. Cells were pelleted and washed in PBS before addition of Hoechst33342 (Invitrogen, H21492). DNA content was then quantified on a Sony MA900 cell sorter.

### *KIT* structural variant and *MITF* mouse models

The *KIT* and *MITF* variants were generated on a C58BL/6 mouse background using CRISPR-Cas9–mediated genome editing. Briefly, the *KIT* deletion allele was generated by inducing flanking double-stranded breaks using two gRNAs: 5′-CCCAGGCAGACTTCGTGAAC-CGG-3′ and 5′-TCACGAGTATTCAGTGCGGG-GGG-3′; and a single-stranded oligodeoxyribonucleotide template: 5′-AGAGCCTCTGCTCACAGCAAATAAGCCGGTCACAGGGTATTTGTAAGCAGTTAGTTTCAAAGACAACATTGTGTGGCCGTGACCCCAGGCAGACTTCGTGGCACTGAATACTCGTGACCCTGTTCTGGGAGCTGTGGTGTCCTCGATTCACACAGCCCCAACACTTCCCATTTTCTCTAAGGACGTCCACATGGCTCCAG-3′. The *MITF* T>C variant was generated using gRNA 5′-CCAGTAGTATTAATGGACAA-TGG-3′ with a donor DNA template 5′-TTTTTAAAGGATGAGCTATCAAAGTCAAGCTCACTGTCAGATCAAGGCCAAGTCCCCATTCATCTTTCGTTCCAGTAGTATTAATGGACAGTGGTGTTTCTCTTTCAG-CAATAGGTTAAGAGCTGGA-3′. The gRNA-ribonucleoprotein complexes were microinjected or electroporated into zygotes and transferred to pseudopregnant females as previously described by Desjardins *et al.* (*52*). F0 founders and F1 generation were genotyped by conventional PCR (NEB; as described in the “*KIT* structural variant cell models” section; table S12) and Sanger sequencing (Source Bioscience) using DNA extracted from ear tissue biopsies.
