# Supplementary material for: Structural and epistatic regulatory variants cause hallmark white spotting in cattle
Source: Sci Adv. 2025 Nov 14;11(46):eadt5913. doi: 10.1126/sciadv.adt5913 (PMC12617462; doi:10.1126/sciadv.adt5913)
Supplement: Supplementary file 1 — Figs. S1 to S8 Tables S1 to S13 [file sciadv.adt5913_sm.pdf]

Science Advances
AAAS

Supplementary Materials for

Structural and epistatic regulatory variants cause hallmark white spotting in cattle

Swati Jivanji *et al.*

Corresponding author: Mathew D. Littlejohn, mathew.littlejohn@lic.co.nz

Sci. Adv. **11**, eadt5913 (2025)
DOI: 10.1126/sciadv.adt5913

This PDF file includes:

Figs. S1 to S8
Tables S1 to S13

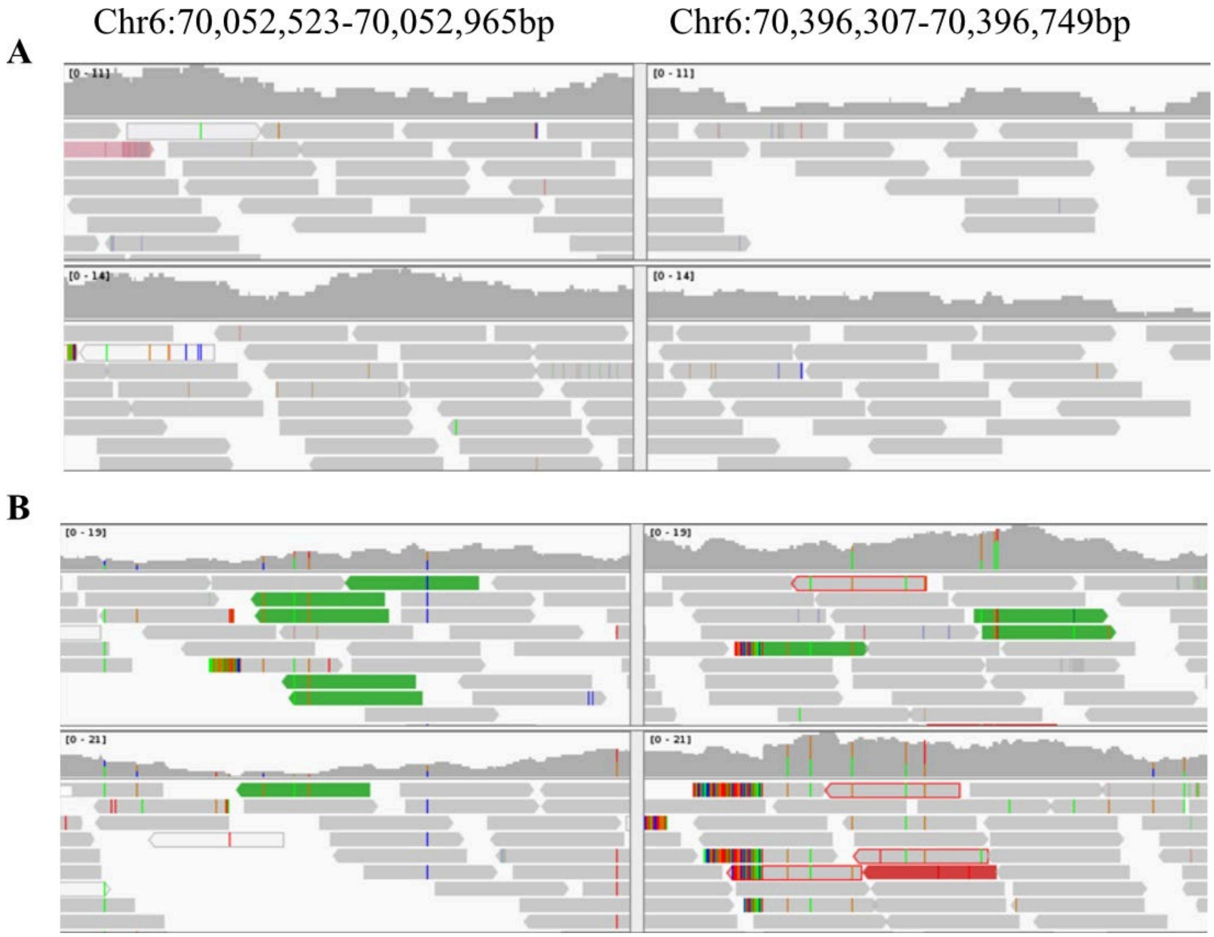

Fig. S1. Sequence alignments across candidate structural variant sites Chr6:70,052,523-70,052,965bp and Chr6:70,396,307-70,396,749bp. (**A**) Sequence alignments from two cattle (one per row) are visualized to demonstrate concordant mate-paired reads. Paired reads map pointing towards each other, where the forward (F) read in the mate-pair is expected to map in a 5' to 3' orientation, and the reverse (R) read is expected to map in a 3' to 5' orientation (pair orientation = F1R2), with an insert size of ~150bp. Read-pairs that meet these criteria appear grey. (**B**) The sequence reads highlighted in green are mate-paired reads that map ~400kbp apart in a discordant R1F2 pair orientation. The string of bases within a sequence read that do not match the reference sequence at the mapped position, referred to as soft-clipped reads, are highlighted as blocks of colored bases at the 5' end of both candidate sites. Reads highlighted with a red outline indicate reads where their mate-paired sequence read had not been mapped.

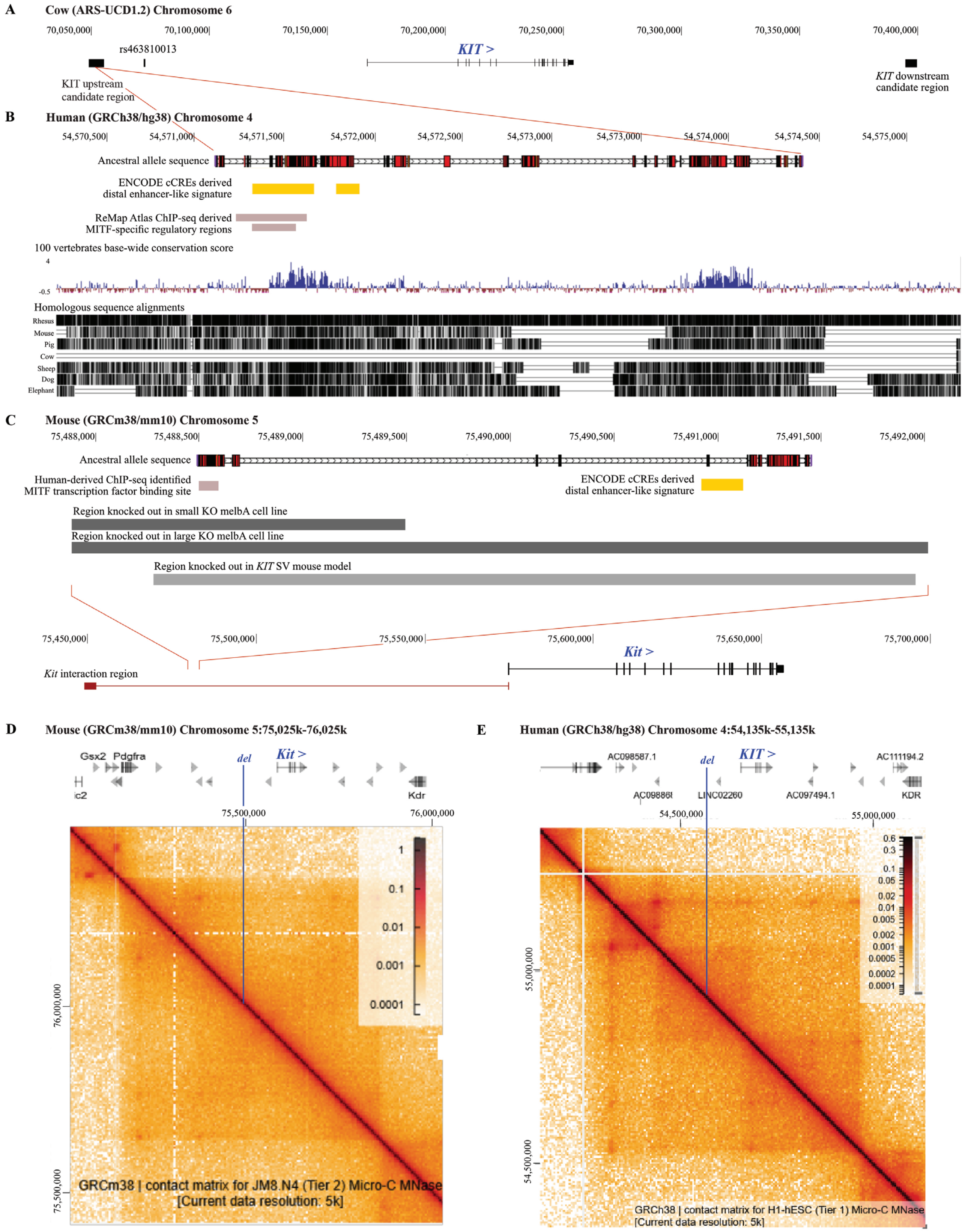

A
Cow (ARS-UCD1.2) Chromosome 6
70,050,000| 70,100,000| 70,150,000| 70,200,000| 70,250,000| 70,300,000| 70,350,000| 70,400,000|
rs463810013
KIT >
KIT upstream candidate region
KIT downstream candidate region
B
Human (GRCh38/hg38) Chromosome 4
54,570,500| 54,571,000| 54,571,500| 54,572,000| 54,572,500| 54,573,000| 54,573,000| 54,574,000| 54,574,500| 54,575,000|
Ancestral allele sequence
ENCODE cCREs derived distal enhancer-like signature
ReMap Atlas ChIP-seq derived MITF-specific regulatory regions
100 vertebrates base-wide conservation score
4
-0.5
Homologous sequence alignments
Rhesus
Mouse
Pig
Cow
Sheep
Dog
Elephant
C
Mouse (GRCm38/mm10) Chromosome 5
75,488,000| 75,488,500| 75,489,000| 75,489,500| 75,490,000| 75,490,500| 75,491,000| 75,491,500| 75,492,000|
Ancestral allele sequence
Human-derived ChIP-seq identified MITF transcription factor binding site
ENCODE cCREs derived distal enhancer-like signature
Region knocked out in small KO melbA cell line
Region knocked out in large KO melbA cell line
Region knocked out in KIT SV mouse model
75,450,000| 75,500,000| 75,550,000| 75,600,000| 75,650,000| 75,700,000|
Kit >
Kit interaction region
D
Mouse (GRCm38/mm10) Chromosome 5:75,025k-76,025k
Gsx2 Pdgfra
del
Kit >
ic2
Kdr
75,500,000
76,000,000
1
0.1
0.01
0.001
0.0001
GRCm38 | contact matrix for JM8.N4 (Tier 2) Micro-C MNase
[Current data resolution: 5k]
E
Human (GRCh38/hg38) Chromosome 4:54,135k-55,135k
AC098587.1
del
KIT >
AC111194.2
LINC02260
AC097494.1
KDR
54,500,000
55,000,000
0.6
0.3
0.1
0.05
0.02
0.01
0.005
0.002
0.001
0.0005
0.0002
0.0001
GRCh38 | contact matrix for H1-hESC (Tier 1) Micro-C MNase
[Current data resolution: 5k]

Fig. S2. Cow, human, and mouse genome-anchored displays showing cross-species alignments and regulatory annotations at the *KIT* locus

A Bovine genomic context of *KIT* structural variant candidate sites relative to the *KIT* gene and GWAS tag SNP rs463810013. **B** Alignment of the bovine 6.9kbp ancestral allele to the human GRCh38/hg38 reference genome, with regulatory and conservation annotations from the UCSC genome browser. The solid black bars of alignment indicate tracts of homologous sequence and red lines indicate variant sites within those sequences. The ancestral allele overlaps ENCODE-annotated distal enhancer-like signatures (indicated in yellow), and ChIP-seq identified MITF transcription factor binding sites (pink segments). This same sequence has a high base-wise conservation score and is highly conserved in a variety of mammals (bottom of **B** panel). Absence of this sequence in cow is due to its absence in the Hereford-derived cow reference genome used for these multi-species alignments. **C** Alignment of the same bovine 6.9kbp ancestral allele to the mouse genome (GCRm38/mm10), with CRISPR-targeted edits for cell models (melbA small and large knockouts), and the live mouse model also shown (dark gray and gray bars respectively). Mouse ENCODE regulatory annotations include a distal enhancer-like signature (yellow), and an enhancer-promoter interacting region that does not overlap the mapped bovine sequence of interest (bottom display, from the EPDnew gene track ENC+EPD). Panels **D** & **E** show Micro-C contact maps for the broader *KIT* locus (1Mbp views). Contact images were extracted from the HiGlass viewer in the 4D Nucleome data portal, and represent Micro-C data generated from mouse JM8.N4 (**D**) and human H1 (**E**) embryonic stem cells (experiment sets 4DNES14CNC1I & 4DNES21D8SP8 respectively). The site of homology to the bovine white spotting alleles is indicated by the blue vertical line within each panel, encompassed within ~730kbp topologically associated domains that appear structurally similar between species.

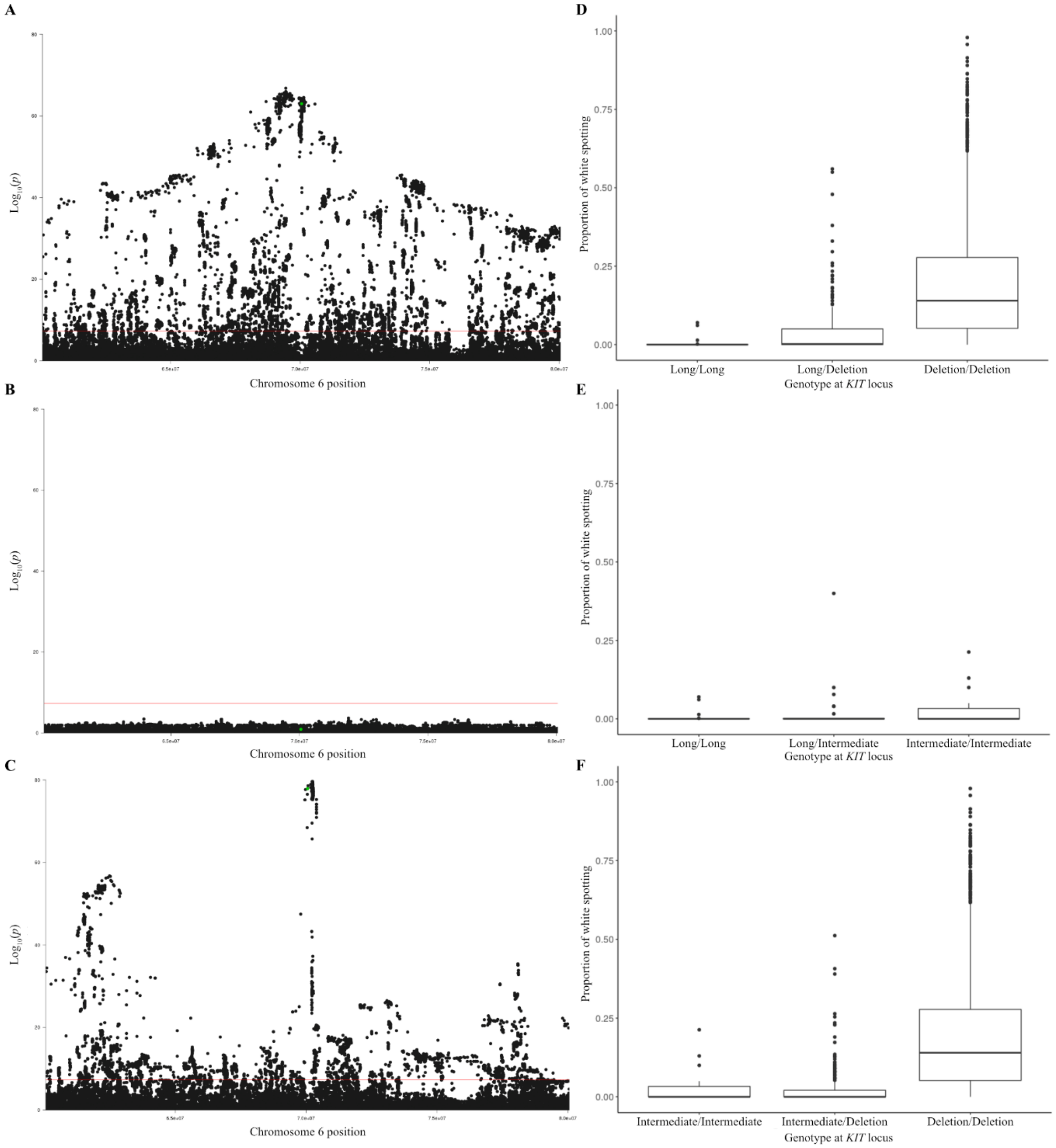

Fig. S3. Association and effect sizes of the *KIT* structural variant states on the proportion of white spotting. (**A**) Manhattan plot based on the association analysis results for the proportion of white spotting in cattle with either the long-form ancestral or deletion alleles. The variant representing the *KIT* structural variant (highlighted in green) has a *p*-value of 1.04×10^{-63}. (**B**) No association signal is seen when contrasting the long-form or intermediate-form ancestral alleles, and (**C**) an association signal is observed when contrasting the intermediate-form ancestral and deletion alleles. The variant representing the *KIT* structural variant (highlighted in green) has a *p*-value of $p=1.25\times10^{-78}$. The red line indicates the significance threshold $p=5\times10^{-8}$. The boxplots show the proportion of white spotting based on genotype at the *KIT* structural variant for cattle (**D**) with the long-form ancestral (Long) or deletion (Deletion) allele, (**E**) with the long-form (Long) or intermediate-form (Intermediate) ancestral allele, and (**F**) intermediate-form ancestral allele (Intermediate) or deletion allele (Deletion).

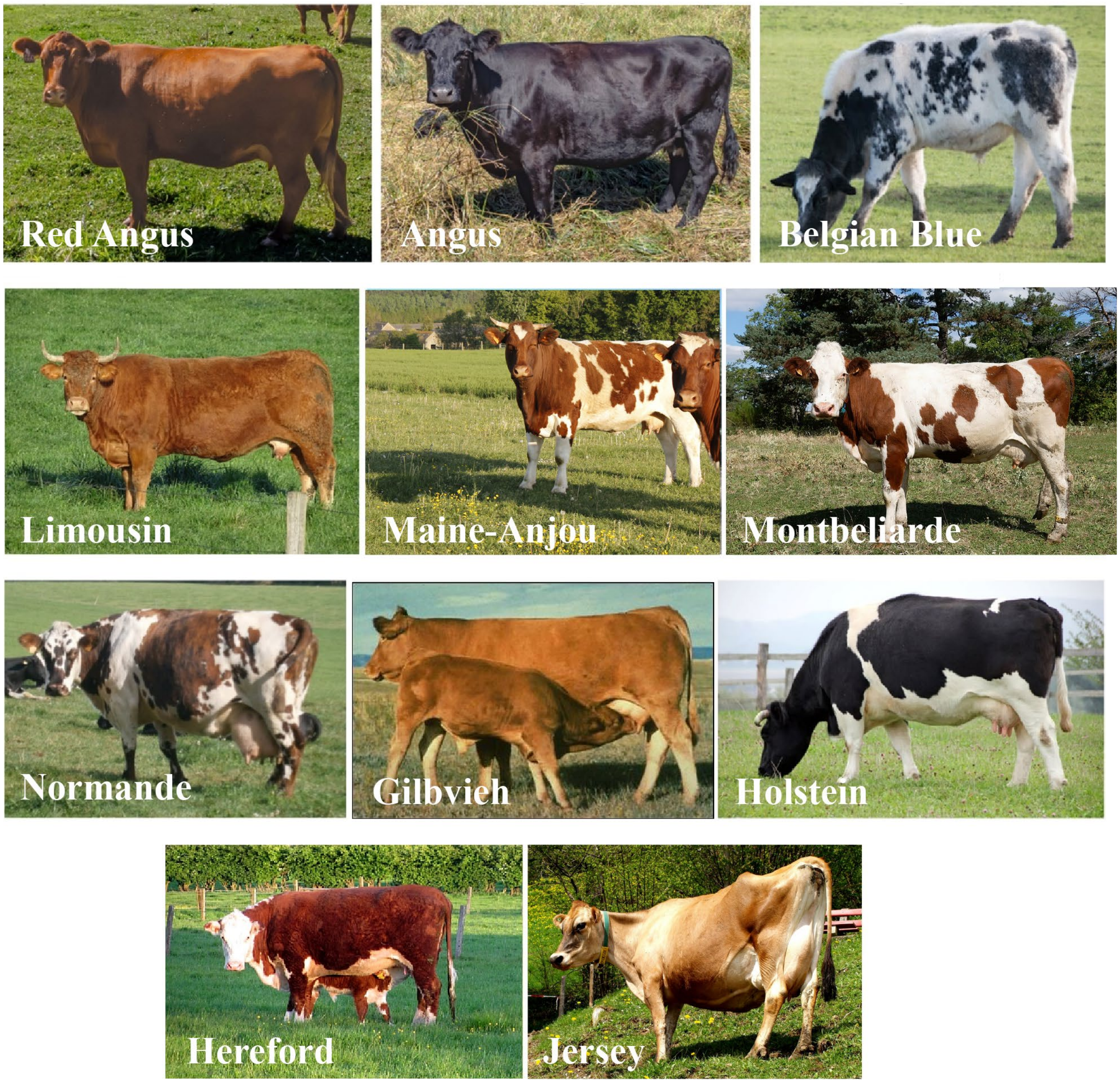

Fig. S4. Figure showing typical coat color and patterning traits observed in Red Angus, Angus, Belgian Blue, Limousin, Maine-Anjou, Montbeliarde, Normande, Gelbvieh, Holstein, Hereford, and Jersey cattle breeds.
Photo credits (from top left): Red Angus – credit anonymous (Wikideas1), https://creativecommons.org/publicdomain/zero/1.0/deed.en. Angus – credit anonymous (Wikideas1), https://creativecommons.org/publicdomain/zero/1.0/deed.en; image mirrored from original. Belgian Blue – credit DawnyH, https://creativecommons.org/licenses/by/3.0/; image mirrored and cropped from original. Limousin – credit Olive Titus; https://creativecommons.org/licenses/by/2.0/deed.en. Maine-Anjou – credit Jean-Bpatiste Bodinier; https://creativecommons.org/licenses/by/2.0/deed.en, image cropped. Montbeliarde – credit Marie-Lan Taÿ Pamart; https://creativecommons.org/licenses/by/4.0/deed.en; image mirrored and cropped from original. Normande – credit Rhian de Kerhiec, https://creativecommons.org/licenses/by/2.0/deed.en; image cropped from original. Gilbvieh –

credit Luagh45, https://creativecommons.org/licenses/by/2.0/deed.en. Holstein – credit anonymous, https://creativecommons.org/publicdomain/zero/1.0/deed.en. Hereford – credit Richard Webb, https://creativecommons.org/licenses/by-sa/2.0/, image cropped from original. Jersey – credit anonymous, https://creativecommons.org/publicdomain/zero/1.0/.

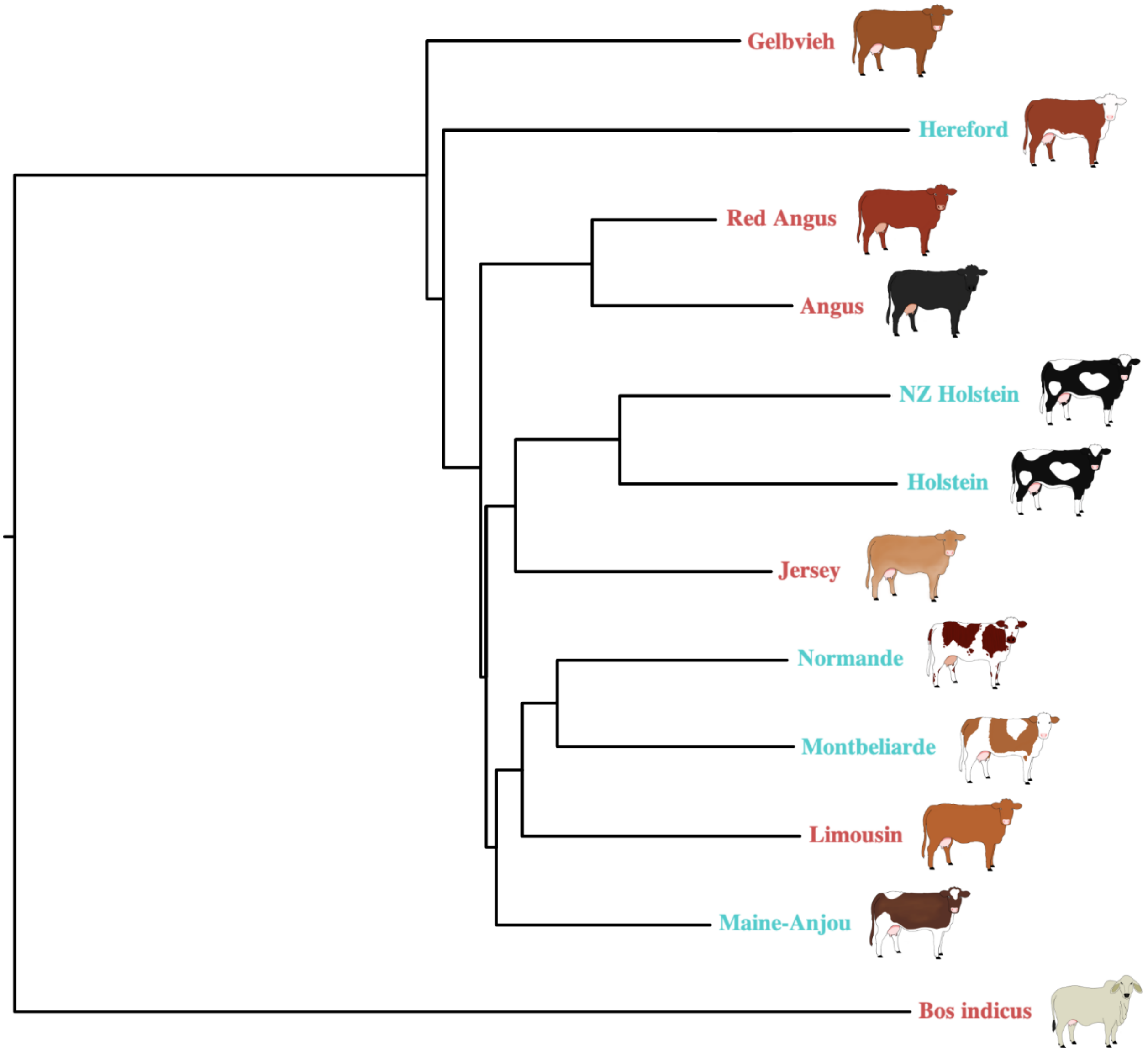

Fig. S5. Mash-based phylogenetic tree for typically spotted (blue) and non-spotted (red) cattle across chromosome 6 constructed using sketch sizes of s=1000, and k-mer sizes of k=21, with Bos indicus included as an outgroup.

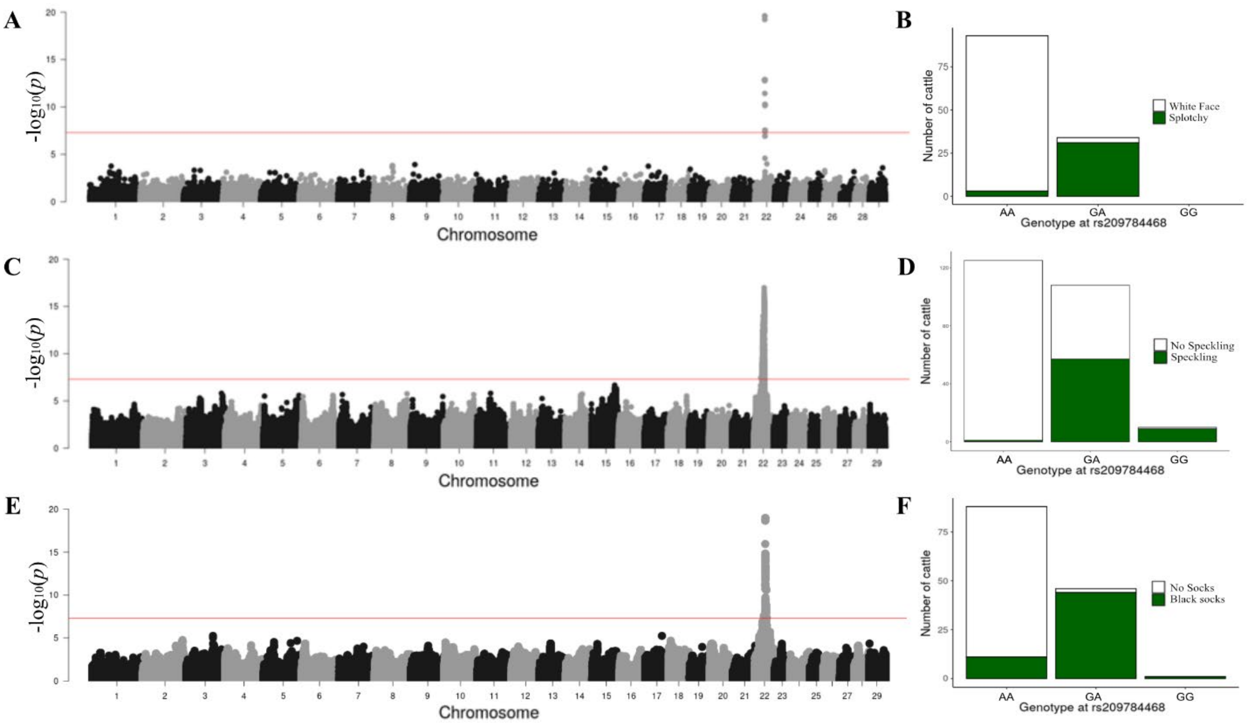

Fig. S6. Association results for splotchy-face, speckling and black socks pattern traits
A Manhattan plot based on association results for the splotchy-face trait. A single signal is observed on chromosome 22, with top variant mapping to Chr22 g.31651404T>C, $p=2.41\times10^{-20}$. **B** The number of cattle with white faces (white) or splotchy faces (green) plotted by their genotype at the candidate causal variant Chr22 g.31651379A>G (rs209784468). **C** Manhattan plot for the speckling trait. A single signal is observed on chromosome 22, with the top variant mapping to Chr22 g.31651379A>G, $p=2.411.04\times10^{-17}$ (the candidate causal *MITF* variant). **D** The number of cattle with no speckles in their white spots (white) or speckles in their white spots (green) plotted by their genotype at the candidate causal variant Chr22 g.31651379A>G (rs209784468). **E** Manhattan plot for the black socks trait. A single signal is observed on chromosome 22, with the top variant mapping to Chr22 g.31650821T>A, $p=1.11\times10^{-19}$. **F** The number of cattle with white legs (white) or black socks (green) plotted by their genotype at the candidate causal variant Chr22 g.31651379A>G (rs209784468).

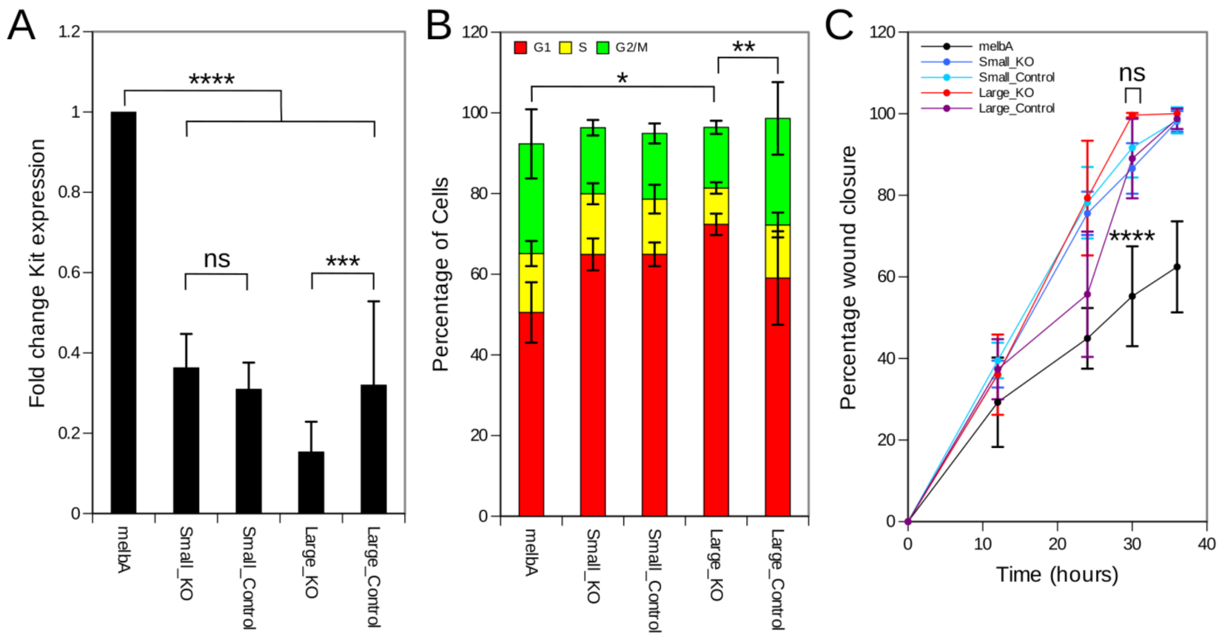

Fig. S7. Heterozygous knockout of the long-form mouse-homologous variant results in reduced *Kit* expression and cell cycle progression in melbA cells. The large and small distal regions to the *Kit* gene were deleted with CRISPR-Cas9 by inducing flanking double-strand breaks in mouse melbA cells. Deleted (KO) and control cells were cloned and genotyped from the same transfection pools; *Kit* expression in the melbA parental cell line (leftmost bar) shows the impact of the differential culture conditions used for transfected pools. The (**A**) *Kit* expression was reduced between the parental melbA group (N=3) and all cloned cell lines (one-way ANOVA $p<0.0001$, Tukey's honestly significant difference (HSD) test $p<0.0001$ in all cases). *Kit* expression was also reduced between the large KO group (N=9; 3 clones) and the large control group (N=9; 3 clones) - Tukey's HSD $p<0.001$. No difference in *Kit* expression was observed between the small KO (N=9; 3 clones), and small control (N=6; 2 clones) groups - Tukey's HSD $p>0.05$. (**B**) FACS analysis of cell cycle phase distributions using Hoescht staining revealed an increase in the proportion of G1 cells between the large KO (N=8; 3 clones) group and both the parental melbA (N=6) and large control (N=9; 3 clones) groups - one-way ANOVA $p<0.01$, Tukey's HSD $p<0.05$ and $p<0.01$ respectively. The proportion of G1 cells did not differ between the small KO (N=9; 3 clones), small control (N=6; 2 clones), or melbA parental groups - Tukey's HSD $p>0.05$. (**C**) Scratch wound healing assays were conducted to assess cell motility. Increased motility was evident between the parental melbA (N=6) group and large KO (N=9; 3 clones), small KO (N=9; 3 clones), large control (N=9; 3 clones), and small control group (N=6; 2 clones) by 30-hours - two-way ANOVA $p<0.0001$(for interaction), Tukey's HSD $p<0.0001$ in all cases. None of the cloned groups differed in motility from one another by 30 hours - Tukey's HSD $p<0.05$ in all cases. Error bars in **A-C** represent 95% confidence intervals; ns = not significant, ** $p< 0.01$, *** $p< 0.001$, **** $p< 0.0001$.

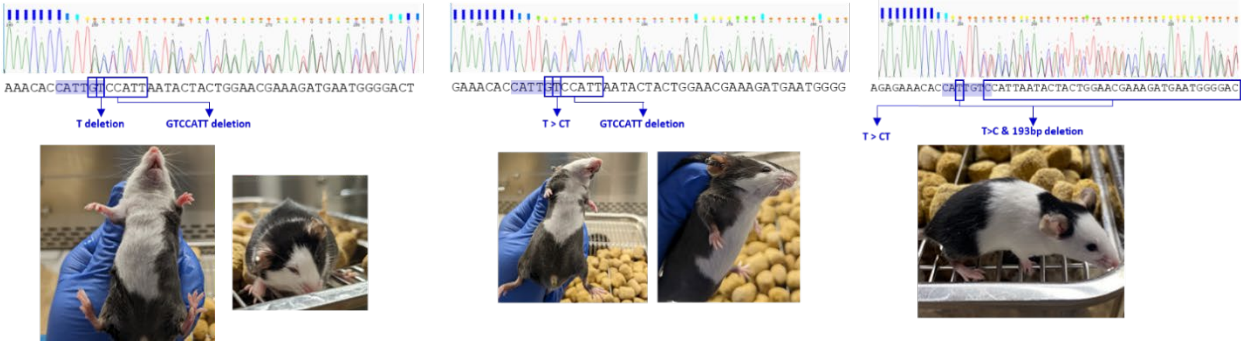

Fig. S8 Phenotypic diversity observed in C58BL/6 mice carrying different off-target edits at the SOX10 transcription factor binding site located to the *Mitf* gene promoter. A range of striking white spotting phenotypes were observed in C58BL/6 mice observed to have off-target edits mapping to, or immediately adjacent to, the SOX10 transcription factor binding site. The off-target variants are shown below the Sanger trace, with the SOX10 transcription factor binding site highlighted in blue. Note all animals shown represent F0 compound heterozygotes.

Table S1. Correlation between inferred structural variant genotypes at Chr6:70,052,523-70,052,965bp (upstream candidate site) and Chr6:70,369,307-70,396,749bp (downstream candidate site) and the proportion of white spotting tag SNP rs463810013.

***KIT* structural variant genotyping method**	**Correlation with rs463810013 (R^2)**
Downstream site genotyped with CNVnator*	0.43
Downstream site genotyped with grep-based method	0.79
Upstream site genotyped with grep-based method	0.87

*Previously reported by Jivanji et al. (9)

Table S2. Additional information on bulls used for long-range PCR and minION sequencing targeting the *KIT* upstream candidate site (Chr6:70,048,910-70,055,246bp) and the downstream candidate site (Chr6:70,394,382-70,399,130bp).

Animal	Genotype at rs463810013	PCR amplicon size		Average minION sequencing depth (×)	
		Upstream site	Downstream site	Upstream site	Downstream site
Jersey 1	TT	13,285bp	4,749bp	5,091	6,822
Jersey 2	TT	13,285bp	4,749bp	4,848	5,098
Jersey 3	TC	13,285bp/6,337bp*	4,749bp	4,799	7,729
Holstein-Friesian 1	CC	6,337bp	4,749bp	7,587	5,711
Holstein-Friesian 2	CC	6,337bp	4,749bp	6,538	6,925
Holstein-Friesian 3	CC	6,337bp	4,749bp	4,487	5,955
Hereford 1	CC	6,337bp	4,749bp	6,769	5,127
Hereford 2	CC	6,337bp	4,749bp	7,974	5,164

* Note amplification bias evident in PCR products from this animal, with the long allele detected via sequencing but not evident from gel electrophoresis.

Table S3. ***KIT*** **structural variant and** ***MITF*** **Chr22 g.31651379G frequencies in spotted and non-spotted cattle breeds.**

Breed	Long-form ancestral allele	Intermediate-form ancestral allele	Deletion allele	Chr22 g.31651379G	Typically spotted or solid coloured
Angus	0.92	0.08	0	0.38	Solid
Red Angus	0.78	0.19	0.03	0.18	Solid
Limousin	0.75	0.25	0	0.60	Solid
Maine-Anjou	0	0	1	0	Spotted
Montbeliarde	0	0	1	0	Spotted
Normande	0	0	1	0.68	Spotted
Gelbvieh	0.78	0.22	0	0.08	Solid
Bos indicus**	0.67	0	0.33	0	-
Holstein	0	0	1	0.01	Spotted
Hereford	0.03	0	0.97	0.01	Spotted
NZ Holstein-Friesian*	0	0	1	0.02	Spotted
NZ Jersey*	0.62	0.13	0.25	0.66	Mostly solid

*Frequencies as derived from reference population and imputed datasets
**Red Fulani and Gudali cattle

Table S4. Top variants mapping to chromosome 6 white-spotting QTL after the *KIT* structural variant was fitted as fixed-effect, with conservation (GERP) score for 91 eutherian mammals (Ensembl Bos taurus v112.13 – ARS-UCD1.3).

Genomic position	*p*-value	GERP Score	Maps to constrained element	Correlation with KIT deletion allele (R^2)	Correlation with Chr6 g.70210094A>C (R^2)
Chr6 g.70343862A>T	8.48×10^{-11}	3.48	yes	0.02	0.02
Chr6 g.70360502A>T	1.88×10^{-8}	-8.01	no	0.02	0.02
Chr6 g.71362789A>G	2.23×10^{-8}	-0.28	no	0.02	0.03
Chr6 g.70359700T>C	2.64×10^{-8}	0.29	no	0.02	0.02
Chr6 g.70360549T>A	2.83×10^{-8}	-2.84	no	0.02	0.03
Chr6 g.70356767A>G	2.83×10^{-8}	-0.46	no	0.02	0.02
Chr6 g.70359256T>C	3.22×10^{-8}	-3.03	no	0.02	0.02
Chr6 g.70360618C>A	3.52×10^{-8}	0.26	no	0.02	0.02
Chr6 g.70359014T>C	3.65×10^{-8}	3.46	yes	0.02	0.03
Chr6 g.70358623T>C	4.23×10^{-8}	0.11	no	0.02	0.02

Table S5. Number of calves included in splotchy face versus white face association analysis by breed composition, phenotype, and genotype at *MITF* candidate causal variant Chr22 g.31651379A>G.

	**Angus × Hereford**		**Holstein-Friesian × Jersey × Hereford**	
**Genotype at Chr22 g.31651379A>G**	AA	GA	AA	GA
White face	8	2	82	2
Splotchy face	0	11	3	20

Table S6. Sequence for 9 probes designed to genotype the *KIT* deletion allele on custom SNP-chip

Probe sequence
TGATCTGAGTACGCAAACTTCTTCCTGAGGTCTGAGAGCCATTCTAGCAGCTTCTCAAGT [C] CAAGGAAAAGGTCATGGAAACCCTGGATTTATACATGACAAGTCAGAAGCACAGGTGCCA
AGTTCACGAGAATTTTTAAGCAGTTACTTAAAAAGACAGCATCGCGTGACCCTAGGAAGG [C] TGCATAAACCCCAGCATGTAGGAATCTTGAGATAAGCTTTCCATTTTACTGAGCACTGCT
AAAGACAGCATCGCGTGACCCTAGGAAGGCTGCATAAACCCCAGCATGTAGGAATCTTGA [G] ATAAGCTTTCCATTTTACTGAGCACTGCTCAACCGCTTCCTAGAATATTGTGTTTGAGCC
CTAGGAAGGCTGCATAAACCCCAGCATGTAGGAATCTTGAGATAAGCTTTCCATTTTACT [G] AGCACTGCTCAACCGCTTCCTAGAATATTGTGTTTGAGCCCTGTAATTAAAGAGGGAAGA
ATTTAGACTGAAGGACAGAGGGTCAAGGAGCAGAGTAACAGTGATCCTCAAACACACTCA [C/T] TCTGGTGTCTGAGAACAGGGCTAATTCTTCTCTGATAAAATAACCACTTGGGGCCCAGCT
AGAACAGGGCTAATTCTTCTCTGATAAAATAACCACTTGGGGCCCAGCTGCTCTCTCTGT [A/G] TGGACTTGTTAGCAAAGAAGACAGAATTCAGCAAGAGGAAGGAGTCGAGTGGTCGGGCTG
CAAGTATCAAAACTGCTGAGGAATAAGAGAGGGGAAACCTTCCCCAGTCCACCATACAAA [G/A] ACATTCACATTAGCATGTATGTCTATGATTTTTCAATTTTTTCACCTATTTTAATGAAGT
TTCTCCAAATGAATATATTTCACTTTTGAACAAAGAAGATGGACACTATTGCACAGCTAT [A] ACGTAACAATTCCCTGAATCCGTATGAACTCTGTGACCATACTGTTTCTAATGGCTATTT
CATTAAGACCCTGATTCAAACAGATTTCTCACTGCTGAAATTCATGATCTGGGCCAAGGG [A/G] TCCCCAGTTTTTGCAGAGTTGTGCTATTGTTGGTTTTGGTGATAACCCCCATTCTATGCA

Table S7. Number of bulls included in speckled versus not speckled association analysis by breed composition, phenotype, and genotype at *MITF* candidate causal variant Chr22 g.31651379A>G.

	Jersey			Holstein-Friesian × Jersey		
**Genotype at Chr22 g.31651379A>G**	AA	AG	GG	AA	AG	GG
No speckles	6	8	1	117	43	0
Speckles	0	15	7	1	42	2

Table S8. Association testing of chr6 and chr22 tag-variants for 23 lactation and animal performance traits

Phenotype		Chr6:71722665<br>Minor allele: T<br>Major allele: C<br>MAF: 0.145			Chr22:31769747<br>Minor allele: G<br>Major allele: A<br>MAF: 0.267		
	n	Effect	Std. Error	P-value	Effect	Std. Error	P-value
Fat yield*	38085	0.0022	0.0070	0.7500	-0.0030	0.0054	0.5900
Protein yield*	38085	0.1007	0.1786	0.5700	0.1154	0.1379	0.4000
Lactose yield*	38085	-0.0001	0.0029	0.9800	-0.0013	0.0022	0.5600
Fat %*	38085	0.0922	0.1151	0.4200	0.1018	0.0889	0.2500
Protein %*	38085	0.0032	0.0017	0.0610	0.0007	0.0013	0.5900
Lactose %*	38085	0.0917	0.1801	0.6100	0.1946	0.1391	0.1600
Milk volume*	38085	0.0072	0.0375	0.8500	0.0405	0.0289	0.1600
Body weight (kg)	76063	-0.4034	0.2875	0.1606	-0.8248	0.2467	8.3E-04
Stature (cm)	72559	0.0024	0.0056	0.6701	-0.0054	0.0048	0.2596
Cond. Score (score)	72926	-0.0059	0.0025	0.0182	-0.0029	0.0021	0.1745
Adaptability (score)	66802	0.0052	0.0102	0.6095	-0.0006	0.0088	0.9451
Temperament (score)	66802	0.0013	0.0097	0.8903	-0.0076	0.0083	0.3592
Farmer opinion (score)	66802	-0.0056	0.0092	0.5465	0.0014	0.0079	0.8602
Chest capacity (score)	72559	0.0011	0.0071	0.8768	0.0078	0.0061	0.2033
Rump angle (score)	72559	0.0073	0.0054	0.1740	-0.0114	0.0046	0.0130
Rump width (score)	72559	-0.0016	0.0059	0.7810	-0.0002	0.0051	0.9633
Leg angle (score)	72559	0.0094	0.0046	0.0408	0.0138	0.0039	4.3E-04
Udder support (score)	72560	-0.0149	0.0074	0.0448	0.0034	0.0064	0.5926
Front udder (score)	72560	0.0051	0.0079	0.5129	0.0169	0.0067	0.0125
F teat position (score)	72560	0.0046	0.0051	0.3704	0.0057	0.0044	0.1912
R teat position (score)	72560	-0.0004	0.0070	0.9546	0.0070	0.0060	0.2432
Udder overall (score)	72560	-0.0064	0.0077	0.4091	0.0060	0.0066	0.3612
Conformation (score)	72559	-0.0075	0.0071	0.2899	-0.0050	0.0061	0.4103

* Estimates from analyses performed in Tiplady *et al.* 2021 (46); all other estimates from phenotypes reported in Reynolds *et al.* (28)

Table S9. Description of cattle populations used for analyses in this study, their sequencing or genotyping platforms and data availability.

Analysis	Population size	Number of cattle per breed		Sequencing/ genotyping platform	Data availability
Discovery dataset	565	Holstein-Friesian	116	Illumina HiSeq 2000 paired-end sequencing	NCBI Sequence Read Archive: SRP276986; previously described by Reynolds et al. (28)
		Jersey	95		
		Holstein-Friesian × Jersey	354		
Imputation reference dataset	1,126*	Holstein-Friesian	280	Illumina HiSeq 2000 paired-end sequencing	NCBI Sequence Read Archive: PRJNA1306123
		Jersey	188		
		Holstein-Friesian × Jersey	659		
KIT SV lab-based characterisation	8	Holstein-Friesian	3	Illumina HiSeq 2000 paired-end sequencing & Oxford nanopore minION	NCBI Sequence Read Archive: PRJNA1295666
		Jersey	3		
		Hereford	2		
Proportion white association analysis	2,976**	Holstein-Friesian	592	Imputed to sequence	https://doi.org/10.5061/dryad.tqjq2bvtf; previously described by Jivanji et al. (9)
		Jersey	274		
		Holstein-Friesian × Jersey	2,110		
Phylogenetic comparison	548	Angus	82	Illumina HiSeq 2000 paired end sequencing	
		Red Angus	29		
		Charolais	29		

		Limousin	10	&	NCBI Sequence Read Archive accession
		Maine-Anjou	7		identifiers: ERP010431, SRP017441, and
		Montbeliarde	17	Illumina HiSeq 4000 paired	SRP245473
		Normande	17	end sequencing	
		Gelbvieh	30		&
		Bos indicus	3		
		Holstein	73		NCBI Sequence Read Archive accession
		Hereford	35		identifier: SRP276986; previously described
		NZ Holstein-Friesian	120***		by Reynolds et al. (28)
		NZ Jersey	96***		
Splotchy face association analysis	128	Angus × Hereford	21	SNP chip genotypes and phenotypes	Genotype and phenotype information uploaded to https://doi.org/10.5061/dryad.fxpnvx15q
		Holstein-Friesian × Jersey × Hereford	107		
Speckly association analysis	242	Jersey	37	Illumina HiSeq 2000 paired end sequencing; genotypes and phenotypes	NCBI Sequence Read Archive accession identifier: SRP276986; previously described by Reynolds et al. (28). Genotype and phenotype information uploaded to https://doi.org/10.5061/dryad.fxpnvx15q
		Holstein-Friesian × Jersey	205		

Black socks association analysis	135	Belgian Blue 135	Imputed sequence data	Genotype and phenotype information uploaded to https://doi.org/10.5281/zenodo.15629509

*Includes all cattle from the discovery dataset

**Includes 499 cattle from the discovery dataset

***Derived from the discovery dataset

Table S10. Twenty-nucleotide search strings used to initially genotype cattle for the upstream (Chr6:70,052,523-70,052,965bp) and downstream (Chr6:70,369,307-70,396,749bp) candidate sites.

	Upstream site	Downstream site
Search string with split-reads	GGTCGCATTCATAGAAACATAGAAACTAGGTGAAG-TGTGT	GAGGGGATATATGTATACCGTGAACTTCCTGATG-TTGAAG
Reverse complement	ACACACTTCACCTAGTTTCTATGTTTCTATGAATGCG-ACC	CTTCAACATCAGGAAGTTCACGGTATACATATAT-CCCCTC
Reference	AGAAAGATGTTAACAGTAGGAGAAACTAGGTGAAG-TGTGT	CTTCAGCAATAAGTGAACCATGAACTTCCTGATG-TTCAAG
Reverse complement	ACACACTTCACCTAGTTTCTCCTACTGTTAACATCTT-TCT	CTTGAACATCAGGAAGTTCATGGTTCACTTATTG-CTGAAG

Table S11. Long-range PCR primer sequences and expected amplicon sizes for targeted amplification of the upstream and downstream candidate sites.

Targeted region (based on ARS-UCD1.2 reference genome)	Forward primer sequence (5' – 3')	Reverse primer sequence (5' – 3')	Expected amplicon size based on reference
Chr6:70,048,910-70,055,246bp	CTGAAGAAGGTGGGAAGGGTTT	CTGGAGAGAGGAGAGTGCAATG	6,337bp
Chr6:70,394,382-70,399,130bp	CTTGGGATCTAACACAGGCCAT	CAGCACAGATTCAGCCACATCT	4,749bp

Table S12. Oligonucleotide sequences used for *KIT* structural variant cell model.

**Oligonucleotides used to generate CRISPR/Cas9 gRNA constructs (5' – 3'):**	
Common_gRNA_For	caccgCCTCGGAGTCATTAACGGAG
Common_gRNA_Rev	aaacCTCCGTTAATGACTCCGAGGc
Large_gRNA_For	caccgCCAAAGTGTGCCCTAACGTGTTC
Large_gRNA_Rev	aaacGAACACGTTAGGGCACACTTTGGc
Small_gRNA_For	caccgCCTTATGGTACCGCTTTCACAGT
Small_gRNA_Rev	aaacACTGTGAAAGCGGTACCATAAGGc
**Oligonucleotides used for screening and sequencing (5' – 3'):**	
Large_For	GGGCTGTCCTGAAGGTTCCAG
Large_Rev	GAAGCCATCAGCTGCATCTG
Small_For	GGCTGTCCTGAAGGTTCCAGC
Small_rev	GCGACTGTGTCAGCGACTGCG
Wt_For	GGGCTGTCCTGAAGGTTCCAG
Wt_Rev	GGAAGCGCTCGAGCAGCTCC
**Oligonucleotides used for qPCR (5' – 3'):**	
β-actin_For	CCACCATGTACCCAGGCATT
β-actin_Rev	CGGACTCATCGTACTCCTGC
cKit-For	CCAACTTCGCCTGACCAGAT
cKit_Rev	AATCCCTCTGCCACACACTG

Table S13. PCR primer sequences, expected amplicon sizes, and targeted regions for targeted amplification of CRISPR-Cas9 targeted editing sites in C58BL/6 mice.

Variant	Targeted region (based on GRCm38/mm10 reference genome)	Forward primer sequence (5’ – 3’)	Reverse primer sequence (5’ – 3’)	Amplicon size
KIT SV	Chr5:75488278-75491958	CCCAGCAACACACCCCTAAC	GCTAAAACATCATGAACCCCAGG	646bp
MITF T>C	Chr6:97991232-97991784	ATTAGTACCCCTCCCCCACC	CCGGAGACGTTATCACAGCA	553bp
MITF wild-type	Chr6:97991232-75488799	ATTAGTACCCCTCCCCCACC	CCAGGCCCCAAATGGTTTTTA	462bp
